# An Ultrasensitive Mechanism Regulates Influenza Virus-Induced Inflammation

**DOI:** 10.1371/journal.ppat.1004856

**Published:** 2015-06-05

**Authors:** Jason E. Shoemaker, Satoshi Fukuyama, Amie J. Eisfeld, Dongming Zhao, Eiryo Kawakami, Saori Sakabe, Tadashi Maemura, Takeo Gorai, Hiroaki Katsura, Yukiko Muramoto, Shinji Watanabe, Tokiko Watanabe, Ken Fuji, Yukiko Matsuoka, Hiroaki Kitano, Yoshihiro Kawaoka

**Affiliations:** 1 ERATO Infection-induced Host Responses Project, Japan Science and Technology Agency, Saitama, Japan; 2 Division of Virology, Department of Microbiology and Immunology, Institute of Medical Science, University of Tokyo, Tokyo, Japan; 3 School of Veterinary Medicine, Department of Pathobiological Sciences, Influenza Research Institute, University of Wisconsin-Madison, Madison, Wisconsin, United States of America; 4 Laboratory for Disease Systems Modeling, RIKEN Center for Integrative Medical Sciences, Kanagawa, Japan; 5 Department of Emerging Infectious Diseases, Institute of Tropical Medicine, Nagasaki University, Nagasaki, Japan; 6 Laboratory of Veterinary Microbiology, Department of Veterinary Sciences, University of Miyazaki, Miyazaki, Japan; 7 Tokyo Metropolitan Institute of Medical Science, Tokyo, Japan; 8 The Systems Biology Institute, Tokyo, Japan; 9 Okinawa Institute of Science and Technology, Okinawa, Japan; 10 Department of Special Pathogens, International Research Center for Infectious Diseases, Institute of Medical Science, University of Tokyo, Tokyo, Japan; Harvard Medical School, UNITED STATES

## Abstract

Influenza viruses present major challenges to public health, evident by the 2009 influenza pandemic. Highly pathogenic influenza virus infections generally coincide with early, high levels of inflammatory cytokines that some studies have suggested may be regulated in a strain-dependent manner. However, a comprehensive characterization of the complex dynamics of the inflammatory response induced by virulent influenza strains is lacking. Here, we applied gene co-expression and nonlinear regression analysis to time-course, microarray data developed from influenza-infected mouse lung to create mathematical models of the host inflammatory response. We found that the dynamics of inflammation-associated gene expression are regulated by an ultrasensitive-like mechanism in which low levels of virus induce minimal gene expression but expression is strongly induced once a threshold virus titer is exceeded. Cytokine assays confirmed that the production of several key inflammatory cytokines, such as interleukin 6 and monocyte chemotactic protein 1, exhibit ultrasensitive behavior. A systematic exploration of the pathways regulating the inflammatory-associated gene response suggests that the molecular origins of this ultrasensitive response mechanism lie within the branch of the Toll-like receptor pathway that regulates STAT1 phosphorylation. This study provides the first evidence of an ultrasensitive mechanism regulating influenza virus-induced inflammation in whole lungs and provides insight into how different virus strains can induce distinct temporal inflammation response profiles. The approach developed here should facilitate the construction of gene regulatory models of other infectious diseases.

## Author Summary

Vaccines suffice for protecting public health against seasonal influenza viruses, but when unexpected strains appear against which the vaccine does not confer protection, alternative treatments are necessary. In this work, we used gene expression and virus growth data from influenza-infected mice to determine how moderate and deadly influenza viruses may invoke unique inflammatory responses and the role these responses play in disease pathology. We found that the relationship between virus growth and the inflammatory response for all viruses tested can be characterized by ultrasensitive response in which the inflammatory response is gated until a threshold concentration of virus is exceeded in the lung after which strong inflammatory gene expression and cytokine production occurs. This finding challenges the notion that deadly influenza viruses invoke unique cytokine and inflammatory responses and provides additional evidence that pathology is regulated by virus load, albeit in a highly nonlinear fashion. These findings suggests immunomodulatory treatments could focus on altering inflammatory response dynamics to improve disease pathology.

## Introduction

Invading pathogens induce acute inflammation when molecular signatures are detected by pattern recognition receptors (PRRs; e.g., RIG-I like receptors [RLRs] and Toll-like receptors [TLRs]) expressed on tissue-resident immune cells and non-immune cell types. PRR ligation triggers innate immune responses and leads to the induction of inflammatory and antiviral gene expression, which together function to limit pathogen growth, activate the adaptive immune response, and ultimately resolve the infection [1,2]. Precise regulation of PRR-mediated signaling is necessary to both avoid inadvertent tissue damage in response to non-pathogenic stimuli, and to prevent immunopathology resulting from excessive expression of inflammatory molecules. In essence, the ideal inflammatory response must exhibit a balance between appropriate activation against a genuine threat and self-limiting behavior once that threat has been controlled. Despite its importance in maintaining normal tissue homeostasis and limiting pathogen-associated diseases, the mechanisms underlying the regulation of this balance are poorly understood.

Influenza A viruses are recognized by both TLRs and RIG-I-like receptors (RLRs) [3–7], and some strains are potent inducers of inflammatory and antiviral gene expression. Generally, lung tissues infected with pathogenic isolates exhibit high virus titers and robust inflammatory gene expression, as has been documented in *in vivo* studies with the 1918 Spanish influenza virus [8,9], highly pathogenic H5N1 avian influenza viruses [10–12], and the 2009 H1N1 pandemic influenza virus [13,14]. In contrast, seasonal influenza viruses typically replicate less efficiently, elicit more restrained inflammatory responses, and are usually not associated with lethal infections. Recent evidence has implicated the level of virus replication in infected lung tissues as the primary phenotypic variable driving inflammation and lethal outcomes [15,16]. Other data indicate that influenza viruses that exhibit significant differences in pathogenicity stimulate qualitatively similar host responses that differ primarily at the level of magnitude and kinetics [17]. However, these studies have not revealed the mechanisms that account for the different profile dynamics observed in infections by high and low pathogenic viruses. Such information would aid in clarifying not only how some influenza viruses induce lethal disease, but also the general mechanisms that regulate inflammatory balance.

To characterize the dynamics of influenza virus-induced inflammation, we developed a novel approach to infer gene regulatory models from dynamic gene expression data. Referred to as systems inference microarray analysis, our method builds on current approaches that use co-expression analysis to isolate modules of functional signatures in gene expression data and then extends these methods by fitting the gene expression modules to mathematical equations (models) by using segmented regression analysis. Models can be created to look for strain-dependent responses and, unlike traditional differential expression analysis, to predict gene expression under new experimental conditions. By using this method, we set out to determine how influenza viruses that exhibit variable pathogenicity profiles influence the dynamics of the inflammatory response.

## Results

### H1N1, pH1N1, and H5N1 have distinct virulence and growth dynamics

**Fig 1:**
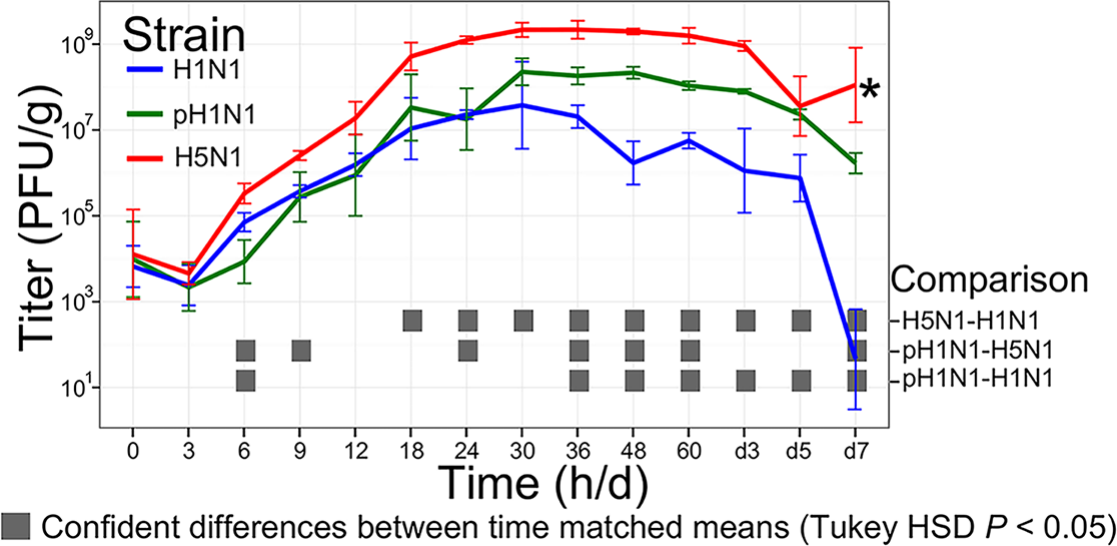
Virus growth dynamics. Mice were infected with 10^5^ PFU of H1N1, pH1N1, or H5N1 virus, three mice per infection group were euthanized at 14 time points after infection (0, 3, 6, 9, 12, 18, 24, 30, 36, 48, and 60 h and 3, 5, and 7 days), and virus titers in lung tissues were determined by plaque assays in MDCK cells. Error bars illustrate the standard deviation from the mean. The gray boxes at each time point identify significant pairwise differences between the means of the viruses indicated at the right of the figure panel (significance was determined by ANOVA followed by Tukey’s Honestly Significantly Different test, *P* < 0.05). *, three H5N1-infected mice intended for collection on day 7 succumbed to their infections prior to sample collection.

**Fig 2:**
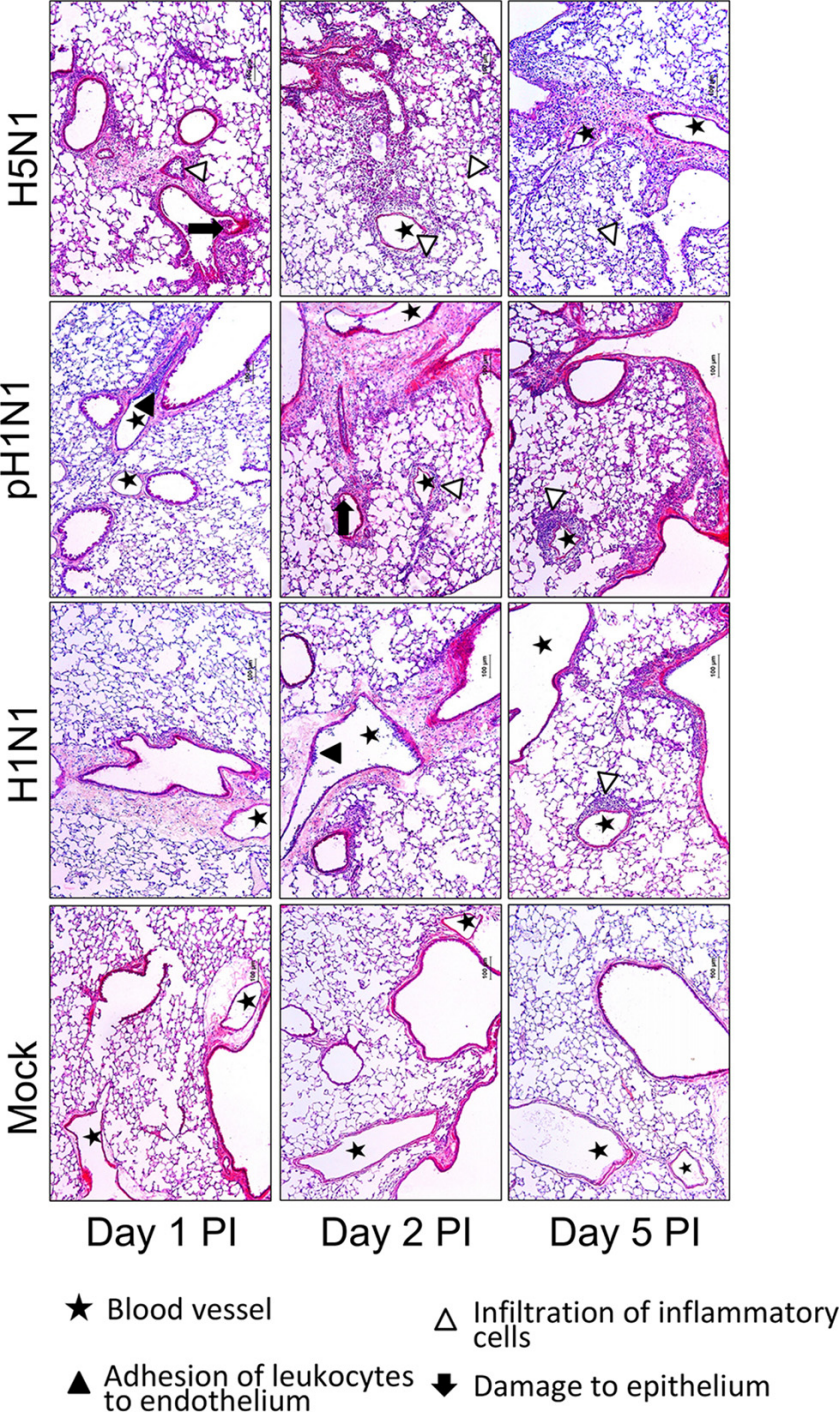
Representative pathological images of influenza-infected lungs. Symbols, as described by the key, emphasize regions of leukocyte adhesion, infiltration of inflammatory cells, and damage to the respiratory epithelium.

To characterize the dynamics of the host immune response to specific virus isolates, we infected mice with 10^5^ PFU of three virus isolates with distinct pathogenicity profiles and harvested lung tissues at 14 time points after infection (from 0 to 7 days post-infection; n = 3 per virus per time point; see Fig 1) for several parallel analyses. These viruses included a low pathogenicity seasonal H1N1 influenza virus (A/Kawasaki/UTK4/2009 [H1N1]; referred to as ‘H1N1’), a mildly pathogenic virus from the 2009 pandemic season (A/California/04/2009 [H1N1]; referred to as ‘pH1N1’), and a highly pathogenic H5N1 avian influenza virus (A/Vietnam/1203/2004 [H5N1]; referred to as ‘H5N1’). An initial inoculation of 10^5^ PFU was used as previous studies indicated that a high virus dose was needed to invoke different pathologies in H1N1 and pH1N1-infected mice [13]. As expected, lung virus titers (virus titers determined by plaque assay and reported in plaque forming units [PFU] per gram lung; Fig 1) indicated a clear hierarchy of mild, moderate, and severe virus-induced disease. Specifically, the H5N1 virus produced the highest lung titers and between days 5 and 7 post-infection, this virus also caused mortality in the animals whose lungs were to be collected on day 7 post-infection (i.e., ‘severe’ disease). In contrast, all animals infected with the H1N1 or pH1N1 viruses survived the duration of the time course study; however, pH1N1-infected animals were visibly sicker and exhibited higher lung titers relative to those infected with H1N1 at all time points observed after the first 30 h post-infection (i.e., ‘moderate’ and ‘mild’ disease, respectively). Histopathological analysis of tissue samples collected on days 1, 2, and 5 post-infection (Fig 2) also showed that H5N1-infected tissue exhibited the earliest, most severe signs of inflammation and inflammatory immune cell infiltrates followed by pH1N1-infected tissue, whereas H1N1-infected tissue showed mild evidence of inflammation and was most similar to tissue from the control mice (mock-infected mice).

### Inflammation-associated gene co-expression is associated with inflammation pathway signaling and macrophage infiltration

We next used co-expression analysis to integrate inflammation-associated gene expression differences between influenza-infected and control lungs into a systems level context. We first asked whether the expression of inflammation-associated genes clustered into modules of co-expressed genes. Tissues from the same animals that were used to determine virus growth were used to evaluate changes in global lung transcriptional profiles. A total of 168 microarrays were developed (three per time point for H1N1-infected, pH1N1-infected, H5N1-infected and control mice). One microarray was removed after reviewing replicate quality. After filtering transcripts for minimally confident variation (we required at least one time-matched, infected condition compared with mock-infected absolute fold change ≥ 2 and a false discovery rate [FDR]-adjusted *P*-value < 0.01), the log_2_ of the normalized intensity of the retained transcripts (16,063) for all 167 samples were then clustered by using the Weighted Gene Co-expression Network Analysis (WGCNA) algorithm [18]. In all, 45 distinct co-expression modules were identified (referred to as N1, N2, etc.; S1 Table provides the module assignments for all transcripts). To identify the biological role of the host response modules, we performed functional enrichment analysis on each gene module by using DAVID [19] and ToppCluster [20]. Because each module was comprised of positively and negatively correlated transcripts, we used the module eigengene (i.e., the first principle component of the gene expression matrix) to divide each module into two submodules containing transcripts that were positively or negatively correlated with the parent module’s eigengene, denoted as kME+ and kME- (referred to as module membership), respectively. This procedure allowed us to look for biological processes with similar but opposing dynamic responses to the virus infections. Functional enrichment analyses were then applied to each submodule by using two bioformatics platforms to ensure robust results.

We identified two submodules (N1 kME+ and N2 kME-, referred to as simply N1 and N2 in the remainder of the text) that were enriched for inflammatory response and inflammation-associated pathway signatures by using both bioinformatics platforms, and these two modules became the focus of our study (Table 1 summarizes the functional enrichment results for the immune and inflammatory related annotations. The complete enrichment results from ToppCluster and DAVID are available in S2 Table and S1 and S2 Files). The N1 module was uniquely enriched for cytokine activity and type I interferon (IFN) regulating TLR and RLR pathways [21], as well as transcriptional signatures associated with IFN-regulated activity (i.e., the transcription factor binding sites [TFBS] of Irf1, Irf7, Irf2, ISRE, and NF-κB). Additionally, N1 was the only module that exhibited significant enrichment with a compendium of established IFN-stimulated genes (‘ISGs’; Table 1; see Methods. The list of ISGs is available in S3 File). A more recent study identified 147 IFN stimulated genes in immortalized, human airway epithelial (Calu3) cells [22]. Of these, 90 mouse homologs were annotated on the microarrays and 70 of the homolog probes were assigned to the N1 module (Fisher’s exact test; *P*-value < 10^–16^; odds ratio = 36.4), further associating N1 interferon-stimulated gene activity.

**Table 1:** Descriptions of immune and inflammation associated gene co-expression modules.

Module	Number of Transcripts (kME+/kME-)	DAVID Annotation Clusters		TC Biological Processes		TFBS		ISGs	
		kME+	kME-	kME+	kME-	kME+	kME-	kME+	kME-
N1	1470 (1021/449)	**Inflammatory Response (16.1)**; TLR Signaling, Cytosolic DNA Sensing Pathway, RIG-I like signaling (12.6); cytokine activity (8.5); innate immune response (8.4); chemokine activity (7.4);	Proteinaceous extracellular matrix (4); blood vessel development (3.2)	Response to virus (10); Cytokine production (10); Inflammatory response (10); Cytokine-mediated signaling pathway (10);		IRF (10), IRF7 (10), ISRE (10), IRF2 (4.2), NFkB (3.5)	—	60.5	0
N2	4813 (3086/1727)	Cilium, cell projection part (7.5); respiratory lung development (6.8); oxidation reduction (4.3);	Response to wounding, **inflammatory response (8.4)**; apoptosis (4.3); cytokine activity (4.1); regulation of leukocyte mediated immunity (3.0)		Leukocyte migration (10); Leukocyte activation (10); Lymphocyte activation (10); Regulation of cell activation (10); Lymphocyte proliferation (5.5)	—	—	0	1.7
N22	146 (126/20)	Pos. reg of immune response, antigen processing and presentation (6.7); leukocyte/lymphocyte activation, differentiation (5.3); MHC class II protein complex (4.8)	—			—	—	0	0
N25	135 (133/2)	Regulation of T cell activation, T cell differentiation, IFNG biosynthesis (11.1); IL 17 Signaling Pathway (7.2);	—			ETS2 (4.9), PU1 (2.8), AML (2.5)	—	0	0
N31	103 (103/0)	Immunoglobulin V-set (10.7);	—			—	—	0	0
N35	73 (64/9)	C-type lectin, Natural killer cell mediated cytotoxicity, natural killer receptor Ly49 (7.6);	—			—	—	0	0

Each module was divided into submodules in which each member gene’s expression was positively or negatively correlated with the module's eigengene (denoted kME+ and kME-, respectively). For each module, we provide the number of transcripts assigned to each submodule, the top DAVID annotation clusters for each submodule (parenthesis shows the-log10 of the average enrichment *P* value for all annotations in the cluster), the top enriched biological processes determined using ToppCluster (parenthesis shows the-log10 of the FDR-adjusted *P* value), the enrichment of established transcription factor binding sites in each submodule (TFBS; parenthesis shows the-log10 of the FDR-adjusted *P* value; performed using ToppCluster), and the enrichment score of a set of IFN-stimulated genes (see Methods; enrichment score is the-log10 of the FDR-adjusted *P* value).

**Fig 3:**
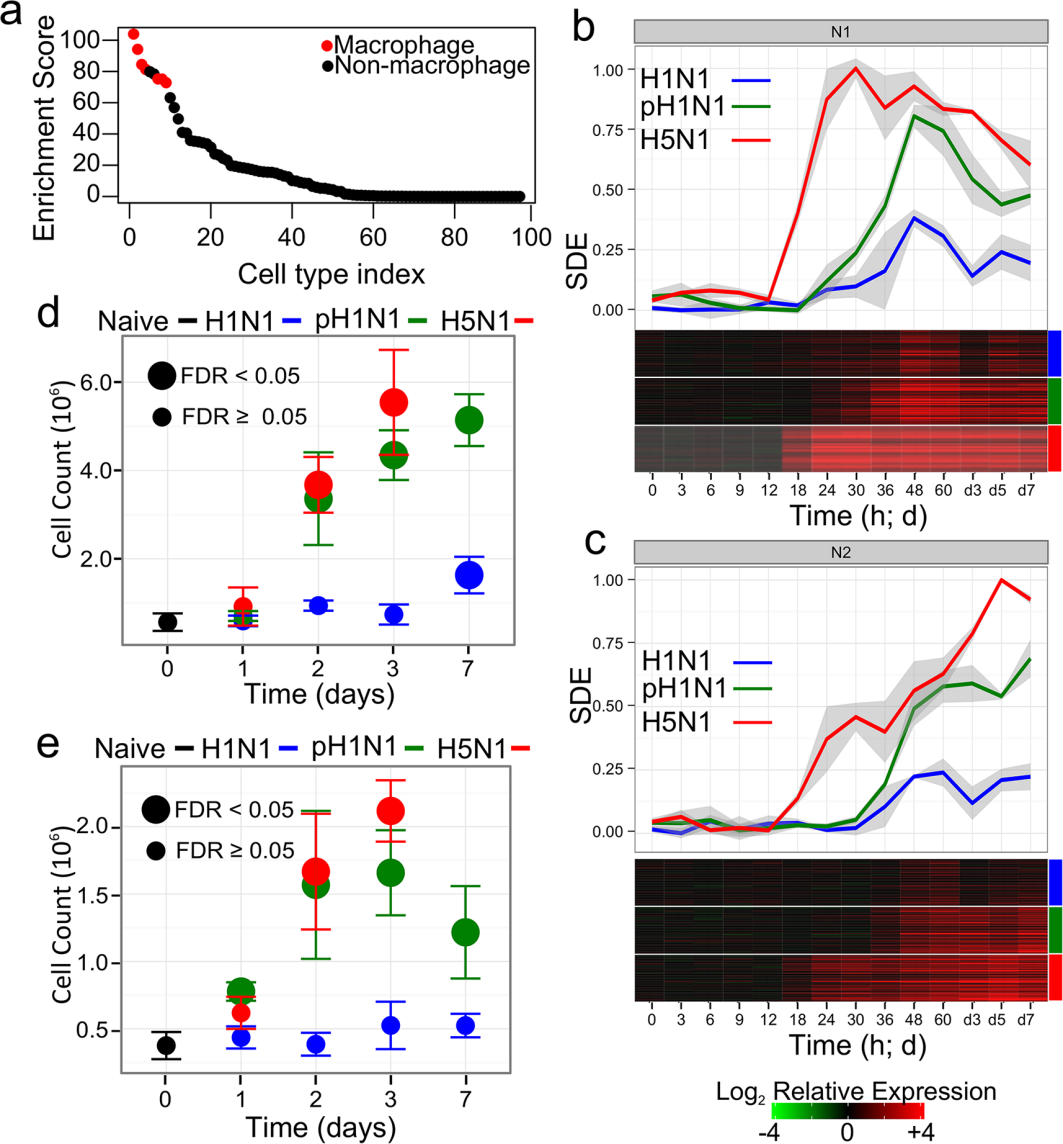
Dynamics of inflammation-associated co-expression modules. Panel (a) shows the enrichment scores (-log_10_ of the FDR-adjusted P value) of the genetic signatures of 94 cell types in the N2 module as determined using CTen[23]. Data corresponding to macrophage signatures in different cellular states are colored red (see S3 Table for the complete results). (b) and (c) The scale difference of the eigengene (SDE) for each influenza virus for the two inflammatory response-related gene co-expression modules N1 and N2, respectively. Different colors denote the specific virus with which the mice were infected, and shaded regions around the plotted data illustrate standard deviations across three biological replicates. A heatmap below each panel shows the gene expression of the top 50 genes (based on their kME) in each module. Color boxes to on the right indicate the specific virus. The fold change is relative to the mean gene expression of pooled, time-matched control samples. Panels (d) and (e) show the cell counts of macrophages and neutrophils in mouse lungs after infection with 10^5^ PFU of influenza viruses (5 mice were used per infection condition at each time point). For each data point, colors above the panel indicate the specific strain. Large points indicate average cell counts that were significantly distinct from the average cell count observed in naïve mice (FDR < 0.05; see Methods).

In contrast, the N2 module was only weakly enriched for some cytokine activity related annotations and not enriched for any of the binding sequences of transcription factors that are members of canonical inflammatory pathways (such as interferons, interferon regulatory factor proteins, or NFκB). Instead, it was primarily associated with several annotations related to leukocyte and lymphocyte activity (see summary of ToppCluster enrichment results in Table 1; see S1 and S2 Files). Further analysis with CTen [23], a platform for associating clustered gene expression data with specific cell types, found N2 to be highly enriched for genes expressed in macrophages in various cellular states (e.g., bone marrow-derived macrophages exposed to lipopolysaccharide [LPS]) (Fig 3A; additional details available in S3 Table). The remaining immune-associated submodules (the kME+ N22, N25, N31, and N35 submodules; described in Table 1) were enriched for several key immune processes such as antigen presentation, and T cell and natural killer (NK) cell activity, but their further assessment would be beyond our focus and the scope of this study. Thus, bioinformatics analyses robustly associated the N1 module with inflammation, cytokine production, and type I IFN pathway activity—likely activated in resident lung cells—whereas the N2 module is associated with migration and activation of macrophages in the lung.

A closer examination of the expression dynamics of each of the inflammation response-associated modules revealed patterns of expression that were consistent with the biological roles predicted by our bioinformatics analyses. We used the scaled difference of the module eigengene to characterize the expression of all genes within each module. We subtracted the mean of the eigengene of the control samples from the eigengene of time-matched, virus-infected samples and then divided by the largest observed average difference across all conditions. The resulting scaled difference eigengene (SDE) represents the fraction of the maximum log fold change in gene expression observed across all experimental conditions (Fig 3B and 3C. S1 Fig briefly illustrates the eigengene scaling in greater detail. S4 Table provides a heatmap of the gene expression of all probes in each module).

Within the inflammation-associated modules (N1 and N2), the H5N1 virus induced the earliest gene expression changes and the highest peak expression levels, corroborating previous observations that H5N1 viruses are strong inducers of inflammatory and IFN response signaling *in vivo* [10,24,25]. Consistent with the prediction that N1 is involved in detecting virus in infected tissues, the N1 module eigengene was the most highly correlated with virus titer (Pearson pairwise correlation, ρ = 0.70).

Module gene expression dynamics further suggested that the N2 module gene is associated with lymphocyte infiltration. Exudate macrophages [26] and neutrophil [27] have been identified as factors of severe disease during influenza infection. To associate gene expression dynamics with changes of immune cell counts, a new population of mice were infected with the three influenza viruses, five mice per infection group were sacrificed on days 1, 2, 3 and 7 and the changes in the number of macrophages and neutrophils was assessed (see Methods). Unlike the previous study by Brandes, *et al*. [27], strong neutrophil infiltration was not specific to fatal infections but, instead, infiltration of both cell types had a clear hierarchical relationship with the severity of the infection (Fig 3D and 3E). The N2 module eigengene exhibited a lesser correlation to virus titer (ρ = 0.55), but was tightly correlated to macrophage influx into the lung (ρ = 0.90; *P*-value < 0.01 Student’s t-test). Of the 45 module identified in the studied, N2 had the highest correlation to both macrophage and neutrophil influx (the correlation of macrophage and neutrophil influx and all 45 module eigengenes are provided in S5 Table; ρ = 0.80; *P*-value < 0.01). The N1 module on the other hand was weakly but significantly correlated with immune cell infiltration (ρ = 0.67 [*P*-value = 0.03] for neutrophils; ρ = 0.60 [*P*-value = 0.05] for macrophages), but its eigengene was not the most highly correlated (5 other module eigengenes had a greater absolute correlation). These results further associate N2 with immune cell-specifically macrophage- infiltration, while the sum of the bioinformatics, virus titer correlations and immune cell infiltration evidences associated N1 with inflammation and type I IFN pathway activity.

### Intramodular network topology suggests Irf7 as N1 module regulator

A further advantage of a network approach is that the functional relevance of genes might be inferred from their positions within the co-expression network [28]. We used the module membership (the correlation between the gene’s expression and the module eigengene, kME) to isolate potential regulators of the N1 module. Among the top intramodular hub genes (i.e., genes with the highest module memberships, see S4 Table), we found *Mnda*, *Herc6* and *Cd274* and several interferon regulated, virus replication inhibitory genes such as *Oas2* and *Oas3* [29]. *Herc6* is involved in ubiquitination [30]. *Mnda* is significantly up-regulated in monocytes exposed to interferon α [31] while *Cd274* is a transmembrane protein expressed on antigen presenting cells and modulates activation of T cells, B cells and myeloid cells. We also observed that interferon stimulated genes tended to have higher intramodular hub rankings, suggesting a regulatory role for interferon (Wilcoxon rank sum test, *P*-value < 10^–12^). We then considered the module membership rankings of transcription factors known to regulate interferon. Of the established interferon regulatory factors that are members of the N1 module (e.g., *Irf1*, *Irf2*, *Irf7*, *Irf9*, *Stat1*, and *Stat2*. *Nfkb1* and *Nfkb2* were not assigned to N1), *Irf1* and *Irf7* had the highest module memberships (kME = 0.94 and 0.93; ranking = 118 and 225, respectively). *Irf7* expression was also several orders of magnitude greater than *Irf1* (S4 Table). Together, these findings corroborate our bioinformatics analyses by suggesting that N1 is regulated by interferon, and N1 expression likely results in enhanced cytopatchic effects and regulation of the lymphocyte immune response. Network analysis further suggests that *Irf7* may play a regulatory role upstream of interferon transcription.

### Inflammation-associated gene expression is regulated by an ultrasensitive mechanism

Previous studies have suggested that highly pathogenic influenza virus infections induce an irregular or disproportionate inflammatory response relative to seasonal influenza viruses, and that these differences occur early in the host response [24]. For this reason, we sought to further explore the possibility of isolate-specific or isolate-independent response patterns of the cytokine-associated N1 module. We wanted to infer mathematical relationships that could describe when inflammatory-associated gene expression occurs and what magnitude of expression is expected. By using the eigengene as a representation of the scaled gene expression dynamics, we attempted to infer simple mathematical models that can be related to common signaling mechanisms.

**Fig 4:**
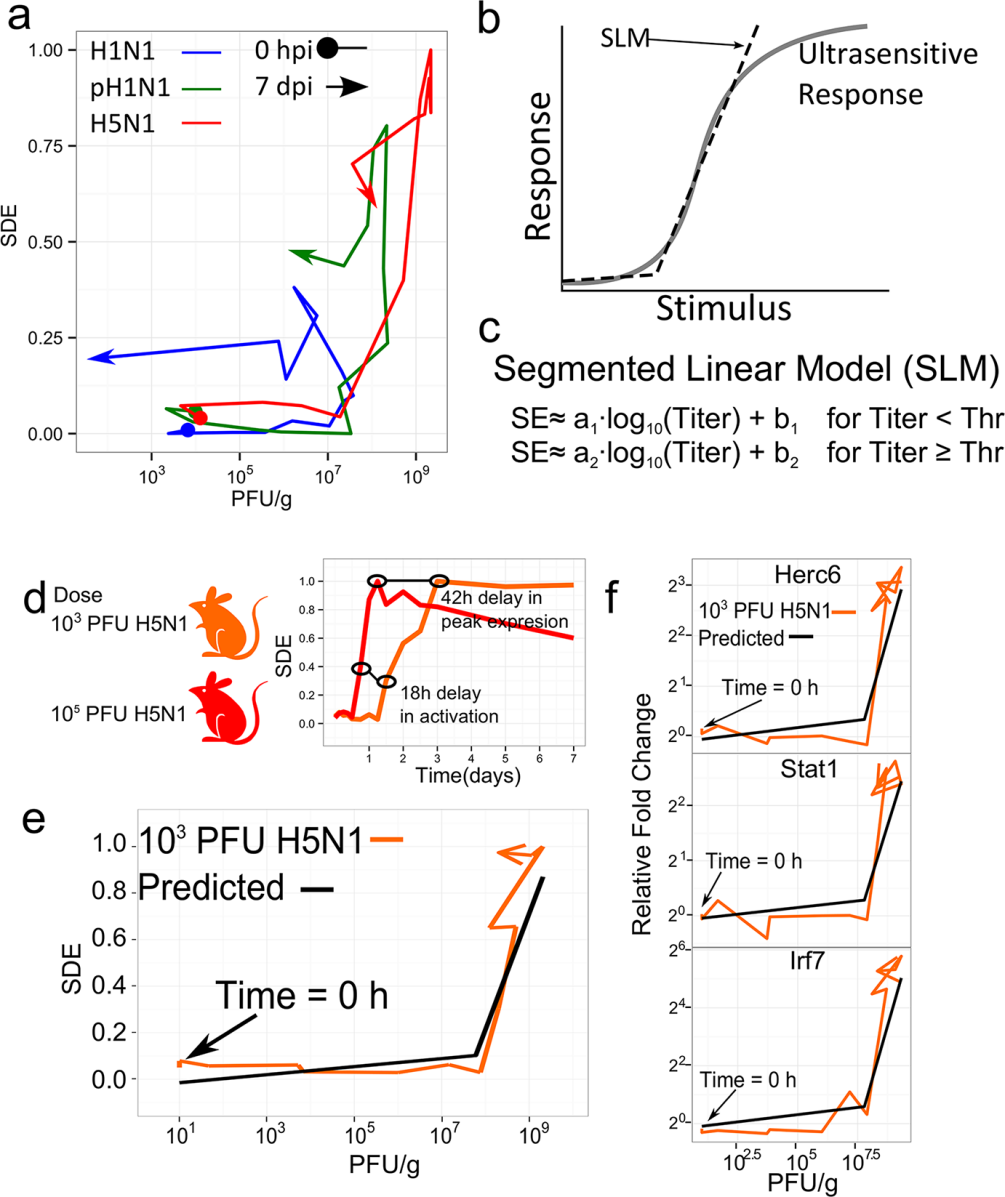
N1 module gene expression is regulated by an ultrasensitive response mechanism triggered by a specific virus concentration. Panel (a) shows a phase-plane representation of the relationship between the scaled N1 module eigengene and the virus titer for each influenza virus infection condition (different colors denote each of the three influenza viruses, which are indicated at the top of the panel. To indicate change in time, a circle indicates data at time 0 hpi and an arrow indicates data at 7 dpi). (b) An example illustration of an ultrasensitive response to increasing stimulus. In our model, the response is the change in inflammatory-associated gene expression and the stimulus is the lung virus titers. A segmented linear model (SLM) can approximate key aspects of the ultrasensitive response. In this study, the response is gene expression and the stimulus is the concentration of virus in the lung (c) The mathematical definition of an SLM. Below some threshold virus titer (Thr), the scaled eigengene (SE) is approximated by a linear model that is a function of the log of the virus titer, the slope (a_1_), and the intercept (b_1_). After the threshold virus titer is reached, the slope and intercept parameters change (a_2_ and b_2_) to describe the SE after activation. The parameters were fit to the data shown in panel (a) and used to predict the N1 SE in newly infected mice. (d) shows a comparison of the H5N1 N1 eigengene from an infection at a dose of 10^5^ PFU (as shown in Fig 3) and an infection at a dose of 10^3^ PFU. Panel (e) compares the predicted scaled eigengene based on the SLM versus the measured eigengene behavior in mice infected with 10^3^ PFU of the H5N1 virus. Panel (f) shows gene expression changes for selected constituent genes of the N1 module as a function of virus titer.

Surprisingly, when we plotted the N1 SDE for each isolate against the corresponding virus titer, we observed a consistent profile for all three viruses; regardless of intrinsic virulence, the fold change in N1 gene expression remained initially low and rapidly increased only after a virus titer of approximately ~10^8^ PFU/g (of lung) was reached (Fig 4A). Following activation, N1 gene expression increased as a function of virus concentration at the same apparent rate for all infection conditions, and more complicated dynamics were observed only during the later phase of the infection when virus clearance was observed (i.e., when the virus titers began to decrease). These observations suggest that IFN-regulated (N1) gene expression was induced by an ultrasensitive response mechanism controlled at the level of the virus titer rather than the virus’s intrinsic virulence.

Ultrasensitive responses characterize the dynamics of several signaling pathways that regulate essential and often toxic biological processes such as the cell cycle [32] and apoptosis [33]. As shown in Fig 4B, ultrasensitive responses are typified by an attenuated response to low levels of stimulation but a strong response occurs once a threshold level of stimulus is reached. Cooperativity [33] and positive feedback [34] are two mechanisms that produce ultrasensitive responses. To formalize the hypothesis that the inflammatory gene response follows an ultrasensitive response profile, we selected a segmented linear model (SLM, defined in Fig 4C) to be a simplified representation of the ordinary differential equations normally used to model ultrasensitive responses, and we fit the N1 SDE to an SLM that was strictly a function of the virus titer. The optimal fit showed a threshold of 10^7.78±0.14^ PFU/g is required for N1 module activation to occur, after which the SDE’s rate of activation (a_2_) was 0.5±0.07 log_10_(PFU/g)^-1^ with an intercept (b_2_) of -3.8 (unitless) (see the Methods and S2 Fig for additional details). Below this threshold, the model predicted minimal gene expression (a1 = 0.17; b1 = 0.03). The SLM goodness of fit on the training data was an adjusted R^2^ = 0.72 while an adjusted R^2^ = 0.41 was observed when the data was fit to a linear model. A Davie’s test confirmed that a segmented model was a significantly better fit than a linear correlation model (*P*-value < 2.2e-16). While the H1N1-infected lung tissue did not exceed an average peak virus titer of 10^7.4^ PFU/g (peak titer occurs at 48 hpi in Fig 4A), we observed increased transcriptional activity in H1N1-infected mouse lung tissues after this time point, suggesting either that the actual peak virus titer occurred between 48 hpi and the subsequent time point (60 hpi), or that the model-predicted threshold was slightly over-approximated.

We next sought to validate the threshold model by attempting to predict cytokine-associated gene expression in influenza virus-infected lung when only the virus titers are known. For this, we selected the H5N1 virus, which has previously been associated with an excessive cytokine response [10]. We infected mice with 10^3^ PFU of the H5N1 virus (a 100-fold reduced dose compared with that used in the experiments to fit the model), determined lung virus titers at the same time points used for the initial experiment (S3 Fig), and then evaluated the segmented linear model’s ability to predict cytokine-associated gene expression. First, we confirmed that the original transcripts assigned to the N1 module were again co-expressed, and thus we used the same transcripts originally assigned to the N1 module to determine the eigengene (see S4 Fig for an analysis of the conservation of the N1 module between the two experiments). In this experiment, as expected, the peak average virus titer (10^9.3±0.21^ PFU/g) for the 10^3^ PFU dose was delayed compared with that for the 10^5^ PFU dose (S3 Fig, compare to Fig 1). Moreover, the SDE exhibited an 18-h delay in activation and a 42-h delay in peak expression compared with the 10^5^ PFU N1 eigengene (Fig 4D). Importantly, based on the virus titers alone, the fitted segmented linear model accurately predicted N1-like SDE behavior (R^2^ = 0.71 for all time points and R^2^ = 0.87 for time points up to peak expression; Fig 4E and S5 Fig), and this could be further demonstrated at the individual gene level for specific N1-associated transcripts (e.g., Herc6, Stat1 and Irf7; Fig 4F). These observations provide strong evidence that activation of inflammatory-associated gene expression is dictated by a specific virus concentration in infected tissue, and further suggest the novel possibility that the pulmonary innate inflammatory response has a nonlinear, ultrasensitive-like activation profile that promotes tolerance to low concentrations of virus.

### The dynamics of key inflammatory cytokines are consistent with an ultrasensitive response

**Fig 5:**
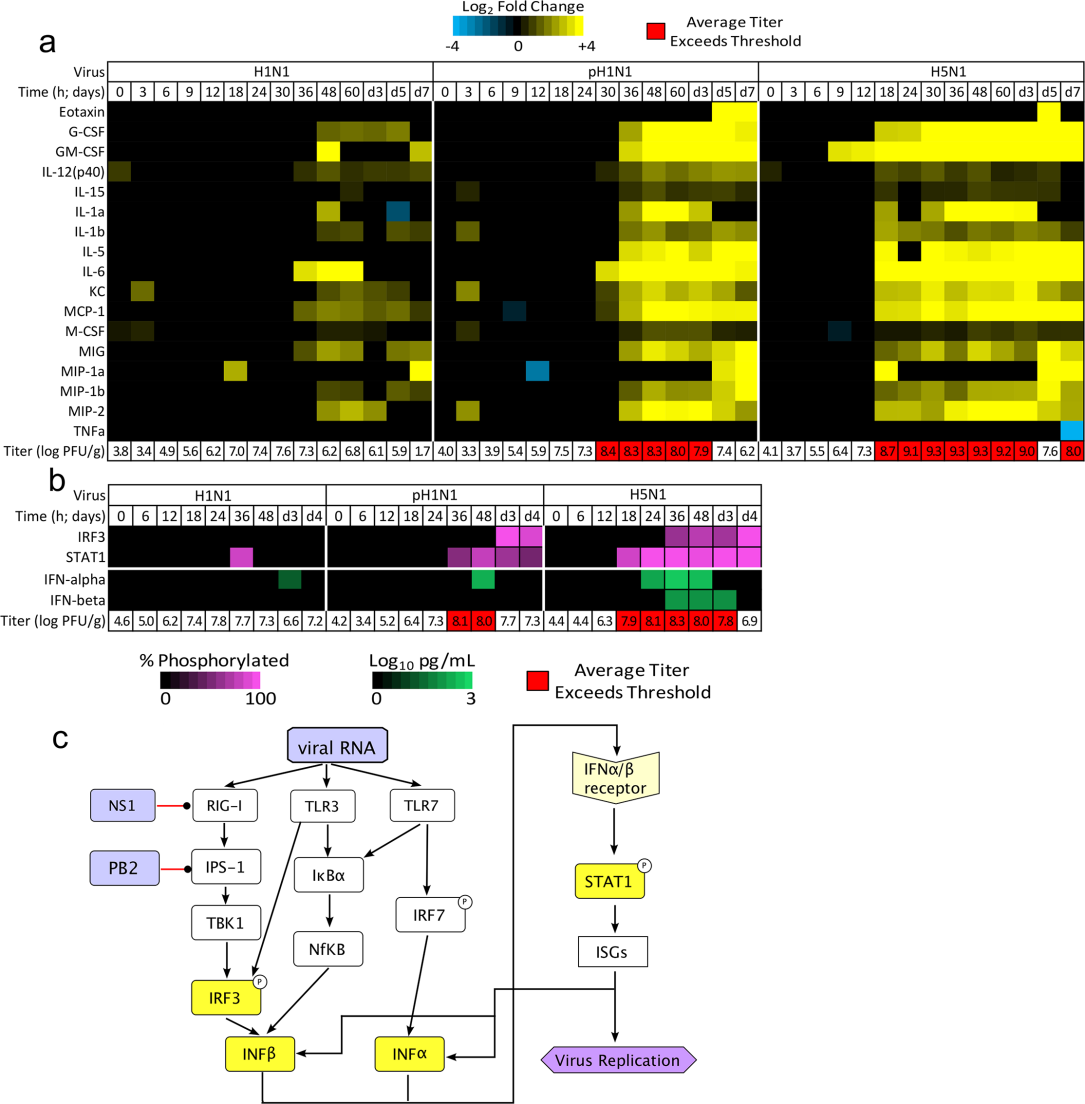
Threshold-like behavior is observed on upstream and downstream components of the IFN signaling pathway. (a) Lung tissues from the same infected mice used for the original gene expression analysis were subjected to cytokine array analysis. Thirty-two cytokine protein concentrations were measured and log scaled (data points lower than the limit of detection for each protein were set to 0.001 to allow scaling), and the average fold change relative to uninfected, time-matched lung samples was determined. The heat map illustrates protein expression values for the 17 cytokines that had transcripts assigned to the N1 module (a blue-to-yellow scale shows expression levels, as indicated by the color key at the top of the panel; time points in which the change in the cytokine levels were not significant [FDR-adjust *P* < 0.05] were set to zero); of these, 15 exhibited expression profiles that were highly significantly correlated with the corresponding transcript and the N1 module eigengene (Pearson’s ρ ≥ 0.4 and FDR < 0.01; eotaxin and TNF-α were the only two that did not exhibit a significant, direct correlation). Non-N1 cytokines are shown in S6 Fig. Virus titers from the same infection are shown below the heat map, with titers surpassing the threshold level identified in the SLM indicated in red. (b) Another set of mice was infected with 10^5^ PFU of H1N1, pH1N1, or H5N1 and lung tissues were harvested (3 mice per infection per time point) for immunoblot (STAT1, phosphorylated STAT1, IRF3, and pIRF3) or ELISA (IFN-α, IFN-β) analysis; virus titers were also determined. The heat map indicates the mean percentage of phosphorylated protein (purple) or the mean interferon concentration (pg/mL; green), and virus titers for each time point are indicated below. Only significant changes are shown in the heat map (FDR-adjusted *P*-value < 0.05 relative to mock-infected samples). Titers highlighted in red indicate that the threshold level (as determined in the initial analysis) was exceeded. Panel (c) shows a schematic of known canonical pathways that regulate IFN production in response to virus infection. Black arrows denote induction whereas red lines denote points at which influenza virus is known to impair these pathways. IFN-α is primarily produced by antigen-presenting cells (APCs) whereas IFN-β is produced in virus-infected, non-APC cells. Both IFNs can induce STAT1 phosphorylation and expression of ISGs in responding cells.

Although transcriptional activation of IFN-stimulated and inflammatory gene expression is a reasonable measure of the effects of inflammation response stimulation, we reasoned that a *bona fide* ultrasensitive mechanism that regulates this response should be reflected in other aspects of the associated signaling pathway(s). Indeed, of the 17 cytokines associated with the N1 module, 15—including key inflammatory proteins, such as interleukin 6 (IL-6) and monocyte chemoattractant protein-1 (MCP-1)—were significantly correlated with the N1 module eigengene (Pearson’s ρ≥0.5; FDR-adjusted *P*-value < 0.01; see S6 Table). In addition, when the protein expression levels of these 15 cytokines were plotted against the corresponding titer data, we observed dynamics similar to that of the inflammatory-associated N1 gene module. Initially, protein expression was low but strongly increased only after the virus titers exceeded the threshold of ~10^8^ PFU/gram determined in the gene regulation model (Fig 5A). In contrast, most of the protein levels of the other measured cytokines with transcripts that were not assigned to the N1 module did not show any obvious relationship to the proposed threshold response (S6 Fig). The only major exceptions were LIF, RANTES, and IL18 (S6 Fig). LIF’s gene transcript was not annotated on the arrays whereas IL18’s transcript was not identified as differentially expressed and therefore was not included in the clustering study. The RANTES transcript was included in the clustering study and assigned by the WGCNA algorithm to N2 although the transcript’s correlation to the N1 eigengene suggests it could also have been assigned to the N1 module (Pearson correlation = 0.84 and 0.89 to the N1 and N2 eigengene, respectively). Some proteins appeared to conform to the threshold model in pH1N1-infected, but not H1N1- or H5N1-infected, mice. These may be cytokines that have strain-dependent responses and do not conform to the model. Overall, changes in N1-associated cytokine protein levels in influenza virus-infected mouse lungs were consistent with the proposed virus-titer regulated threshold-mechanism underlying the IFN-mediated response.

### The molecular origins of the ultrasensitive behavior likely lie in pathways upstream of STAT1

We then searched for evidence of threshold-like behavior in the upstream signaling events leading to the activation of IFN-mediated, inflammation-associated gene expression: namely IFN-α/β protein expression, and IRF3 and STAT1 transcription factor phosphorylation (Figs 5B, S7 and S8. Images of representative blots are available in S9 Fig). Significant increases in the concentration of IFN-α and phosphorylated STAT1 (pSTAT1) were detectable in infections with all three virus isolates and occurred at time points after the threshold level of virus was exceeded (Fig 5B). For the H1N1 data, significant levels of pSTAT1 were observed at 36 hpi which—as noted previously—corresponds to the time immediately after the virus titers in H1N1-infected mice reached their peak (see previous discussion). On the other hand, significant increases in phosphorylated IRF3 (pIRF3) were observed only in pH1N1 and H5N1 infections, whereas significant increases in IFN-β were observed only in the H5N1 infection. Changes in the levels of IFN-β and pIRF3 occurred after significant increases in pSTAT1 and IFN-α occurred. As such, the change in the percentage of pSTAT1 was more closely correlated to the N1 eigengene than was that of pIRF3 (correlation = 0.77±0.06 and 0.67±0.09 respectively), and significant increases in pSTAT1 corresponded to time points at which the mean virus titer exceeded the threshold level identified in the gene expression analysis (~10^7.78^ PFU/g). The greater correlation of IFNα and STAT1 activation to the inflammation-associated, N1 module’s gene expression dynamics and the enrichment of the N1 module for the IRF7 binding sequence (Table 1) suggest that the primary driver of the threshold-regulated, inflammatory gene response originates along the IRF7 → IFN-α → STAT1 axis (Fig 5C).

## Discussion

Our data reveal that the activation of the IFN-associated inflammatory and antiviral response program in influenza-infected mouse lung is characterized by an ultrasensitive response driven by the virus load. The power of the threshold model is illustrated by its ability to accurately predict gene expression in infected mice, and the data further suggest that the molecular basis of threshold behavior originates upstream of IFN-α production. Threshold-like and ultrasensitive mechanisms are hypothesized to be necessary for effective management of critical cellular machinery in noisy environments, and are recognized players in the activation of the cell cycle [34], mitogen-activated protein kinase signaling [35], and apoptosis [33,36]. However, while a role for threshold behaviors have been postulated to be essential for filtering noise or errant signaling in complex biomolecular environments [35], our study is the first to directly link threshold-like behavior to the virus-induced innate immune response.

The ultrasensitive response observed in this study provides additional insight into the mechanisms that drive severe pathologies during influenza infection. Several works have suggested that viral load is a key determinant of pathology [12,15] while other works suggest that highly pathogenic influenza viruses induce early, strong inflammatory responses that are independent of the viral load [9,24,37]. Recently, it was observed that fatal influenza infections in mice coincide with a strong influx of neutrophils in what the author’s describe as a viral load-independent, “feedforward” inflammatory circuit [27]. The ultrasensitive response suggested by our study consolidates these hypotheses by suggesting that viral load drives cytokine production (and in turn immune cell infiltration) in a nonlinear manner which is capable of producing states of high and low innate immune responses. Characterization of key aspects of the inflammatory response, such as the onset and peak inflammatory gene expression, require a high temporal resolution of the virus growth and host response dynamics; an experimental design that was unique to our study. The ultrasensitive response model does not negate the significance of neutrophil infiltration [27] in determining fatal infections but suggests that viral load drives the high and low innate immune states. The observed threshold may represent the transition to immunopathology; as indicated by the histopathology results (Fig 2). Moreover, the influenza virus’ NS1 protein is crucial for inhibiting the interferon-mediated antiviral response [38]. The NS1 protein of three viruses used in this study have the SUMO1 acceptor site that indicates interferon antagonism capability [39–41]. Additional studies with NS1-mutated viruses and other pathogens may better reveal strain-dependencies for the observed thresholding behavior.

The ultrasensitive response further suggests that the innate immune response has a limited capacity to respond to influenza virus infection and supports the development of immunomodulatory therapies. Interestingly, after the threshold was exceeded, the rate of activation for inflammatory and interferon-associated gene expression (N1) was conserved for a moderately pathogenic and deadly viruses (Fig 4A). The conserved rate of activation implies that the immune response detects the virus concentration but not the virus growth rate; suggesting the innate immune response is naturally limited in its ability to respond to high growth influenza viruses. Additionally, studies in knockout mice indicate that type I IFN-associated pathways are essential for protection during primary infection [42] and that earlier initiation of these pathways coincides with increased survival in mice infected with highly pathogenic isolates [43]. In combination with these studies, the findings here suggest a novel means of protecting high risk groups by treating them with compounds that target the molecular mechanisms responsible for the threshold behavior. Lowering the threshold required to induce the cytokine response may be a means of providing protection from severe influenza infection. Since these compounds would target host proteins, such treatments would be effective against various influenza virus strains. Data from the viruses studied here suggest that post-threshold, inflammatory gene expression primarily reflects interferon-regulated tissue damage, but time-course data from additional highly pathogenic viruses are needed to assess the degree to which interferon activity is associated with virus growth suppression.

## Materials and Methods

### Viruses

The A/California/04/09 H1N1 virus (pH1N1) was received from the Centers for Disease Control and Prevention. The A/Kawasaki/UTK-4/09 H1N1 virus (H1N1) served as a reference for a seasonal influenza, whereas a fatal human isolate, A/Vietnam/1203/04 H5N1 virus (H5N1), served a highly pathogenic virus.

### Ethics statement

All mouse experience were performed in accordance to the University of Tokyo's Regulations for Animal Care and Use. These regulations were approved by the Animal Experiment Committee of the Institute of Medical Science, the University of Tokyo (approval number: PA10-13). The committee acknowledged and deemed acceptable the legal and ethical responsibilities for the animals, as detailed in the Fundamental Guidelines for Proper Conduct of Animal Experiment and Related Activities in Academic Research Institutions under the jurisdiction of the Ministry of Education, Culture, Sports, Science and Technology, 2006.

All experiments with H5N1 viruses were performed in biosafety level 3 containment laboratories at the University of Tokyo, which are approved by the Ministry of Agriculture, Forestry, and Fisheries, Japan.

### Mouse experiments

Five-week old C57BL/6J, female mice were obtained from Japan SLC. For all experiments, mice were anesthetized with isoflurane and intranasally inoculated with either 10^3^ or 10^5^ PFU of virus. Initially, 42 mice were inoculated with 10^5^ PFU of the H1N1, pH1N1, or H5N1 virus or mock-infected with PBS (a total of 168 mice). At 14 time points, 3 mice per group were humanely sacrificed and their lungs harvested. The lungs were sectioned and used to assess virus titers (right-upper lobe), cytokine levels (right lower), and the initial gene expression that was used to train the segmented linear model (left-lower). Separately, 42 mice were infected with 10^3^ PFU of the H5N1, sacrificed at the same 14 time points, and lungs sections obtained as described previously to provide the model validation data. The same inoculation method was used for all mice in this study. The numbers of mice used for flow cytometry, western blot, and interferon protein assay experiments are specific in the corresponding sections.

### Cytokine protein assays

For cytokine and chemokine measurements, mouse lungs were treated with the Bio-Plex Cell Lysis Kit (Bio-Rad laboratories, Hercules, CA) according to the manufacturer’s instructions. Concentrations of other cytokines were determined with the Bio-Plex Mouse Cytokine 23-Plex and 9-Plex panels (Bio-Rad laboratories) Array analysis was performed by using the Bio-Plex Protein Array system (Bio-Rad laboratories).

### Virus titers

Virus titers were determined by plaque assay using MDCK cells.

### RNA isolation and oligonucleotide microarray processing

Mouse lung tissues were placed in RNALater (Ambion, CA), an RNA stabilization reagent and stored at -80°C. All tissues were thawed together and homogenized for 2 minutes at 30 Hz using a TissueLyser (Qiagen, Hilden, Germany) as per the manufacturer’s instructions. From the homogenized lung tissues, total RNA was extracted with the RNeasy Mini Kit (Qiagen, Hilden, Germany) in accordance with the manufacturer’s instructions. Cy3-labeled cRNA preparations were hybridized onto Agilent-014868 Whole Mouse Genome 4x44K microarrays for 17 h at 65°C. Feature Extraction Software version 7 (Agilent Technologies) was used for image analysis and data extraction, and Takara Bio provided whole array quality control metrics.

### Microarray normalization, replicate quality, annotation, and statistical analysis

Differential expression was assessed by using a linear regression model. By using the limma package [44] 26 version 3.14.1 from BioConductor, probe intensities were background corrected by using the “norm-exponential” method, normalized between arrays (using the quantile method), and averaged over unique probes IDs. Replicate quality was assessed using hierarchical clustering, resulting in the removal of a single array (the array corresponded to a sample collected at 3 hpi in H5N1-infected mice.) Probe intensities were fit to a linear model that compared data from infected samples to time-matched data collected from uninfected mice. Probes were annotated by matching to the probe names in the mgug4122a version 2.1 mouse annotation database available from BioConductor. All arrays in this study have been deposited on the GEO Expression Omnibus (GSE63786).

### Gene co-expression analysis

Unsigned co-expression networks were constructed by using the blockwiseModules program from the WGCNA package version 1.23.1 [18] in R. The analysis was performed with several different parameterizations to ensure robust clustering. For the results reported in this text, we removed all probes that did not have a confident fold-change greater than 2 (FDR < 0.01) for at least one infected-tissue to control-tissue, time-matched comparison. We then clustered the log_2_ of the normalized intensities for all 167 microarrays (corresponding the three samples for each time-point for each infected or control population with the exception of data from H5N1-infected mice at 3 hpi which had two samples). Based on the scale-free topology characteristics curve, a power of n = 7 with no reassignment after clustering (reassignThreshold = 0) and a maximum cluster size of 6000 probes was used. We generally observed that allowing gene reassignment between the modules led to poorer clustering based on the distribution the gene’s module memberships (the correlation between a gene and the eigengene of the module to which it had been assigned. S10 Fig illustrates the distribution of the gene kMEs for modules N1 and N2). We then repeated the clustering using different powers (ranging from 7 to 11), allowing different cluster sizes, different subsets of the expression data (e.g., clustering data from each infection separately or together), or relaxing the differential expression condition. In all clusterings performed, the two modules discussed in the text were identifiable. Fisher exact tests between each clustering run were used to determine whether the initial modules were significantly conserved under different parameterizations.

We also considered if the N1 module would be isolated when using signed versus unsigned network construction. We constructed a signed co-expression network and found that 92% of the kME+ N1 genes are again clustered and confirmed that the gene expression dynamics were maintained (see S11 Fig).

### Functional enrichment analyses

ToppCluster [20] and DAVID [19] were used for gene ontology and pathway enrichment, and ToppCluster was also used for transcription factor binding site enrichment analyses. DAVID uses clusters of related annotations constructed from several annotation databases (e.g., pathway and gene ontology annotations) to determine the function of a set of genes and scores the enrichment by averaging the unadjusted P-values (determined by Fisher’s Exact test) of the annotations within the cluster. ToppCluster uses hypergeometric tests to determine the enrichment between a set of genes and gene lists contained in 18 categories (databases) detailed in the ToppGene Suite [45]. The databases include cis-regulatory motif data [46,47], referred to as transcription factor binding sites (TFBS) in the text. Both tools were used with their default settings and the gene universe was considered to be all annotated mouse genes. For each module, we considered the enrichment of all genes assigned to the module and the kME+ and kME- subsets. Generally, the enrichment analysis of the whole module gene set reiterated the enrichment results of the kME+ and kME- subsets albeit with slightly lower but still significant enrichment. Since both tools returned similar GO and pathway enrichment results, we summarized the functional and pathway enrichment results in Table 1 using the results from DAVID.

The enrichment of interferon stimulated genes was determined by using a list of interferon stimulated genes from the Interferon Stimulated Gene Database [48] that was downloaded on May 9, 2012 (see S3 File). For each module, all module genes and the kME+ and kME- subsets were tested for enrichment using Fisher’s exact test in R. The p values were adjusted to control the false discovery rate.

CTen [23] was used to determine enriched cell signatures in select co-expression modules. The enrichment score reported is the—log10 of the false discovery rate.

### Segmented model fitting and validation

Model fitting and validation was performed in R using the ‘segmented’ package [49].

### Flow cytometry analysis

Five mice per time point per infection were infected with 10^5^ PFU of the described virus. Five uninfected (naïve) mice served as negative controls. Whole lungs were collected from mice, and incubated with Collagenase D (Roche Diagnostics; final concentration: 2 μg/mL) and DNase I (Worthington; final concentration: 40 U/mL) for 30 minutes at 37°C. Single-cell suspensions were obtained from lungs by grinding tissues through a nylon filter (BD Biosciences). Red blood cells (RBCs) in a sample were lysed with RBC lysis buffer (Sigma). Samples were resuspended with PBS containing 2 mM EDTA and 0.5% bovine serum albumin (BSA), and cell number was determined by using a disposable cell counter (OneCell). To block nonspecific binding of antibodies mediated by Fc receptors, cells were incubated with purified anti-mouse CD16/32 (Fc Block, BD Biosciences). Cells were stained with appropriate combinations of fluorescent antibodies to analyze the population of each immune cell subset. The anti-F4/80 (BM8; eBioscience) antibodies were used. All samples were also incubated with 7-aminoactinomycin D (Via-Probe, BD Biosciences) for dead cell exclusion. Data from labeled cells were acquired on a FACSAria II (BD Biosciences) and analyzed with FlowJo software version 9.3.1 (Tree Star).

### Western blot analysis

Three mice per time point per infection group were infected with 10^5^ PFU of the described virus. The primary antibodies of mouse anti-STAT1 (phospho Tyr701) mAb (ab29045, abcam), rabbit anti-IRF3 (phospho Ser396) mAb (4947, Cell Signaling), and mouse anti–β-actin (A2228; Sigma-Aldrich) were used; the secondary antibodies were HRP-conjugated anti-mouse IgG antibody (GE Healthcare) and HRP-conjugated anti-rabbit IgG antibody (GE Healthcare). Mouse lungs were collected and homogenized with RIPA buffer (Thermo Scientific, Rockford, IL, USA) containing proteinase inhibitor (Roche, Mannheim, Germany) and phosphatase inhibitor cocktails (Sigma-Aldrich, Saint Louis, Missouri, USA). The lysates were then briefly sonicated and centrifuged. Each sample was electrophoresed on sodium dodecylsulfate polyacrylamide gels (Bio-Rad Laboratories, Hercules, CA, USA) and transferred to a PVDF membrane (Millipore, Billerica, MA, USA). The membranes were blocked with Blocking One (Nacalai Tesque, Kyoto, Japan) for 30 min at room temperature, and then were incubated with the primary antibodies overnight at 4° C, followed by the secondary antibodies. They were then washed 3 times with PBS plus Tween 20 (PBS-T) for 5 min and incubated with secondary HRP-conjugated antibodies (as described above) for 30 min at room temperature, followed by three washes with PBST. Specific proteins were detected by using SuperSignal West Femto Maximum Sensitivity Substrate (Thermo Scientific, Rockford, IL, USA). Photography and quantification of band intensity were conducted with the VersaDoc Imaging System (Bio-Rad Laboratories, Hercules, CA, USA). The quantity of target bands from each sample was standardized by their respective β-actin.

### Interferon protein assays

Three mice per time point per infection group were infected with 10^5^ PFU of the described virus. Half the lung of each mouse was dissolved in 1 mL of RIPA buffer. We measured the Interferon-alpha and Interferon-beta by using ELISA kits (#12100, #42400, PBL Assay Science, NJ, USA) according to the manufacturer’s instructions. Plates were read at an absorbance of 450 nm using a Versa Max plate reader (MolecularDevices, Menlo Park, CA).

### Statistical analyses

Additional gene set overlap tests were performed in R with all of the genes annotated on the array as the reference (background) set. Statistical tests to compare means within the western blot, flow cytometry, immune cell count and protein assay data sets were performed in R using the ‘multcomp’ package [50].

## Supporting Information

S1 Fig The scaled difference of the module eigengene. The eigengene is the first principle component (equivalently the first eigenvector) of a matrix of gene expression data. In clustered data in which all genes are highly correlated (as in the case of WGCNA), the eigengene is a scaled approximation of how gene expression changes for all genes in a module (i.e. cluster) across experimental conditions. The eigengene of module N1 is shown in (a) for the 56 experimental conditions considered in this study (4 infection conditions and 14 time points). Each point represents data from a single animal in each experimental condition (3 animals per condition except for H5N1-infected animals at 3 hpi which had 2; total of 167 samples). While the eigengene illustrates the changes in gene expression of module member genes, it is difficult to interpret the changes with respect to the control data. We therefore scaled the eigengene by subtracting from time-matched data the average of the eigengene from mock-infected animals and then dividing by the highest average eigengene for any experimental condition. The scaled difference of the eigengene (SDE) is shown in (b). The SDE now represents the fraction of greatest gene expression (i.e., the fraction of the largest log fold change in gene expression observed between all time-matched, infected-to-control samples). For example, the SDE peaks in experimental condition 50 (H5N1-infected animals at 30 hpi), corresponding to the condition in which the log fold change was greatest for N1 member genes (see Fig 3). The SDE for pH1N1-infected animals at day 3 pi (condition 40) is ~0.5. We would expect the log fold change to be approximately half of that observed in H5N1-infected animals at 30 hpi (see the heatmaps in Fig 3). For completeness, we overlay the mean (lines) and the standard deviation (shaded regions) of the SDE (see panel c) to replicate the results shown in Fig 3.
(TIF)

S2 Fig Segmented linear model training and performance. To create the segmented linear model to describe the relationship between the N1 module eigengene and the virus titer data, we selected all titer data that occurred prior to and included the peak of the eigengene and then scaled the data such that the eigengene was bound between [0,1]. The model was then fit to the scaled data. Panel (a) shows the time points selected as training data from each infection data set (indicated by the orange dots); and panel (b) shows the number of data points available for different ranges of the virus titer (top) and the segmented model's fit to the training data (bottom). In panel (c), we used the residuals (i.e., the difference between the predicted values and the actual values) to compare the SLM’s accuracy to that of a simple linear model. The line is the running average and the shaded region is the 95% confidence interval of the mean. The mean of the residuals of the segmented model was always near zero for the full range of virus titers and, thus, is a better fit than the linear model.
(TIF)

S3 Fig Virus titers in mice infected with 10^3^ PFU of the H5N1 virus. To determine whether the threshold model could predict N1 module gene expression, mice were infected with 10^3^ PFU of the H5N1 virus and lung tissues were harvested for virus titration and gene expression microarray analysis at the same time points used for the original experiment (3 mice per infection group and time point). For each time point, the mean and standard deviation of the virus titer is shown.
(TIF)

S4 Fig Comparison of N1 module-associated transcript behavior between 10^3^ and 10^5^ PFU H5N1 virus infections. Two procedures were applied to determine whether the N1 genes that we identified as co-expressed in the original clustering analysis were also co-expressed in tissue from mice infected with 10^3^ PFU of H5N1 virus. (a) Compares the correlation between all 1,021 N1 gene transcripts and the eigengene (referred to as the kME) in the original gene expression data (10^5^ PFU infection conditions (referred to as ‘10^5^ PFU’)) and the 10^3^ PFU infection condition (referred to as ‘10^3^ PFU’). More than 90% of the genes exhibited a kME > 0.9. (b) The WGCNA algorithm was repeated with all differentially expressed genes (FDR-adjusted *P*-value < 0.01 and fold change > 2 for at least 1 time point) from the 10^3^ PFU H5N1 virus infection, and then the Fisher's exact test was used to identify modules enriched for N1 genes. Of the modules in the newly constructed co-expression network, only one module was enriched with the N1 transcripts. Specifically, of the 826 genes originally assigned to N1, 528 (Benjimini-Hochberg-adjusted *P*-value < 2.2E-16) were differentially expressed and assigned to the same module in the 10^3^ PFU infection condition, as illustrated by the Venn diagram.
(TIF)

S5 Fig The segmented linear model’s prediction performance in mice infected with 10^3^ PFU of the H5N1 virus (H5N1-10^3^ PFU). The H5N1-10^3^ PFU eigengene was constructed using the same set of probes assigned to the N1 module from the 10^5^ PFU infection condition and scaled to between [0,1]; and then the segmented linear model trained to the 10^5^ PFU H1N1, pH1N1, and H5N1 data was used to predict H5N1-10^3^ PFU scaled eigengene values. Panel (a) shows a comparison of the predicted (black) and actual (pink arrows depicting the temporal evolution of the gene response) eigengenes, with error bars illustrating the standard deviation of the eigengene and of the log_10_ of the virus titer. Panel (b) shows how the prediction residuals are distributed over time. For each time point, individual data points (black points) are shown, as well as the average (red points) and standard deviation (gray bars). The greatest deviations occurred at d5 and d7, which was expected as the model was designed only to predict the onset of gene activation and the peak gene expression (peak of the eigengene). On days 5 and 7 post-infection, both the virus titers and gene expression has already peaked and are declining. Panel (c) shows how the prediction residuals are distributed across the spectrum of observed virus titers, as compared to a linear model directly fit to the H5N1-10^3^ PFU N1 eigengene. Individual residuals are indicated by points, and the running average and the 95% confidence intervals are shown by the colored lines and the gray shading, respectively. As for the 10^5^ PFU infection condition, the segmented model performed well across the entire virus titer spectrum, and was significantly better than a linear model fitted directly to the data.
(TIF)

S6 Fig Protein concentrations of non-N1 module cytokines. As described in the Fig 5 legend, cytokine concentrations were assayed in the lungs of mice infected with 10^5^ PFU of H1N1, pH1N1, and H5N1 and compared with those in lung tissues from mock-infected animals. This heat map illustrates protein expression values for non-N1-associated cytokines (14 cytokine expression profiles are shown; IL-12(p70)(78) was not detected at any time point and is not included), with a blue-to-yellow scale indicating expression levels (see the key to the right of the panel). The module to which the protein's mRNA transcript was assigned during clustering is shown on the right hand side of the heat map (proteins whose gene transcripts were not DE are labeled ‘NA’), and the average virus titers are shown below the heat map (red indicates that titers exceeded the threshold concentration predicted by the segmented linear model). While IL-18 and leukemia inhibitory factor (LIF) exhibited protein expression patterns consistent with N1 module behavior, the transcripts mapping to these proteins were not differentially expressed and were excluded from the co-expression network construction.
(TIF)

S7 Fig Virus titer and immunoblot data from an additional infection experiment with 10^5^ PFU of H1N1, pH1N1, or H5N1 virus. As described in the Fig 5 legend (panel b), an additional set of mice was infected with 10^5^ PFU of H1N1, pH1N1, or H5N1 virus to determine total and phosphorylated levels of transcription factors by means of immunoblotting. Here, virus titers (with standard deviation indicated by gray bars) for each infection condition are shown in panel (a), and immunoblot results are shown in panels (b) and (c). For immunoblot analyses, relative protein concentrations were determined by calculating the ratio of the gray intensity of the measured protein (IκBα, total IRF7, total IRF3 [‘IRF3’], phosphorylated IRF3 [pIRF3’], total STAT1 [‘STAT1’], and phosphorylated STAT1 [‘pSTAT1’]) relative to the gray intensity of actin (i.e., the loading control; referred to as the relative signal intensity, RSI) in each tissue sample. A linear model was used to compare the mean RSI of each protein at each time point to the mean RSI measured in uninfected animals (referred to as ‘naïve’), and a significant difference was defined as having a false discovery rate (FDR) < 0.05. The mean RSI of total IRF3, IRF7, and IκBα did not significantly deviate from the naïve data, but pSTAT1, pIRF3 and total STAT1 significantly differed from the naïve data at several time points (significant distinct time points are indicated by large, black dots).
(TIF)

S8 Fig Changes in lung IFN-α and IFN-β protein levels in mice infected with 10^5^ PFU of H1N1, pH1N1, or H5N1 virus. As described in the Fig 5 legend (panel b), mice were infected with 10^5^ PFU of H1N1, pH1N1, or H5N1 virus to determine IFN protein concentrations. The protein concentration of IFN-α/β proteins for each replicate at each time point is shown.
(TIF)

S9 Fig Representative images of western blots. As described in the Fig 5 legend (panel b), mice were infected with 10^5^ PFU of H1N1, pH1N1, or H5N1 virus. Representative images of Western blots of lung homogenates from 3 mice per infection per time point are shown.
(TIF)

S10 Fig Boxplot of the absolute kMEs of all transcripts mapped to the N1 and N2 modules. The intramodular correlation (kME or correlation between each transcript and the eigengene) can be used to assess clustering quality. We show boxplots to illustrate the distribution of the kMEs for all transcripts belonging to the N1 and N2 modules.
(TIF)

S11 Fig Applying signed co-expression network analysis also clusters the N1 kME+ transcripts. The N1 module presented in this work was identified by developing an unsigned co-expression network and then focusing on the kME- and kME+. Therefore, the N1 kME+ set of genes should also cluster when developing a signed co-expression network. We confirmed this by repeating the WGCNA procedure for signed network (power = 14). Of the N1 kME+ transcripts, 93.2% were assigned to the same cluster in the signed co-expression network (the new module is referred to as *N1signed*). Furthermore, 90.9% if the *N1signed* transcripts were N1 kME+ transcripts. We then confirmed that the same eigengene dynamics were observed. The scaled difference of the eigengene (SDE) of the *N1signed* module is shown versus time (a) and versus virus titers (b).
(TIF)

S1 File Each of the kME+ submodules, labeled N1, N2,…,N45 were analyzed using DAVID bioinformatics tool to determine enriched biological functions. For each module, we report the top enriched annotation clusters as well as various measurements of enrichment (the Bonferroni, Benjamini, and the false discovery [FDR] adjusted p value. Additional information on the enrichment statisticis can be found at david.abcc.ncifcrf.gov
(XLS)

S2 File Each of the kME- submodules, labeled N1, N2,…,N45 were analyzed using DAVID bioinformatics tool to determine enriched biological functions. For each module, we report the top enriched annotation clusters as well as various measurements of enrichment (the Bonferroni, Benjamini, and the false discovery [FDR] adjusted p value. Additional information on the enrichment statistics can be found at david.abcc.ncifcrf.gov
(XLS)

S3 File A list of interferon stimulated genes received from the Interferon Stimulated Gene Database. (XLSX)

S1 Table The co-expression module to which each transcript was assigned. For each transcript we provide the Entrez Gene ID, the gene symbol, the module it was assigned to and the correlation between the transcript's expression dynamics and the expression dynamics of its assigned eigengene (kME). Module N0 is the set of transcripts which the algorithm did not identify as co-expressed.
(XLSX)

S2 Table Enriched annotations identified using ToppCluster. **Each kME+ or kME- submodule was analyzed in ToppCluster for enriched domain, GO biological process, GO molecular function, GO cellular component, mouse phenotype, pathway or transcription factor binding site annotations.** Columns A-D provide information on the annotation, including the category to which the annotation belongs, the database specific annotation ID and the full title of the annotation. Columns D—AT contain the enrichment score for each annotation in each submodule. The enrichment score is the—log10 of the false discovery rate [FDR]-adjusted p value. A threshold enrichment score of 2 (FDR-adjusted p < 0.01) was required to be considered as significantly enriched.
(XLSX)

S3 Table CTen analysis of N2 module. The probes assigned to module N2 were analyzed in CTen—a platform for identifying the genomic signatures of select cell type in microarray data. The enrichment score reported for each cell type is the-log10 of the false-discovery adjusted p value.
(XLSX)

S4 Table A heatmap of the gene expression of the probes assigned to the N1 and N2 modules. Arrays were developed from the lungs of mice infected with H1N1, pH1N1, or HPAI virus at 14 time points. One array from the HPAI infected data set was removed due to quality concerns. For each transcript, we provide annotation information (Probe Name, Systematic Name, Entrez ID, Gene Symbol, and Gene Name), identify to which module the transcript was assigned, the kME (the Pearson correlation coefficient between the transcripts expression and the eigengene of the module it was assigned to), and the log2 fold change in the transcript’s expression across all arrays used the study is illustrated by a heatmap.
(XLSX)

S5 Table Correlation between the changes macrophage and neutrophil cell counts and each module eigengene. (XLSX)

S6 Table Correlation between changes in cytokine protein levels and cytokine gene transcript levels. (XLSX)
