# Supplementary figures and images for: An Ultrasensitive Mechanism Regulates Influenza Virus-Induced Inflammation
Source: PLoS Pathog. 2015 Jun 5;11(6):e1004856. doi: 10.1371/journal.ppat.1004856 (PMC4457877; doi:10.1371/journal.ppat.1004856)

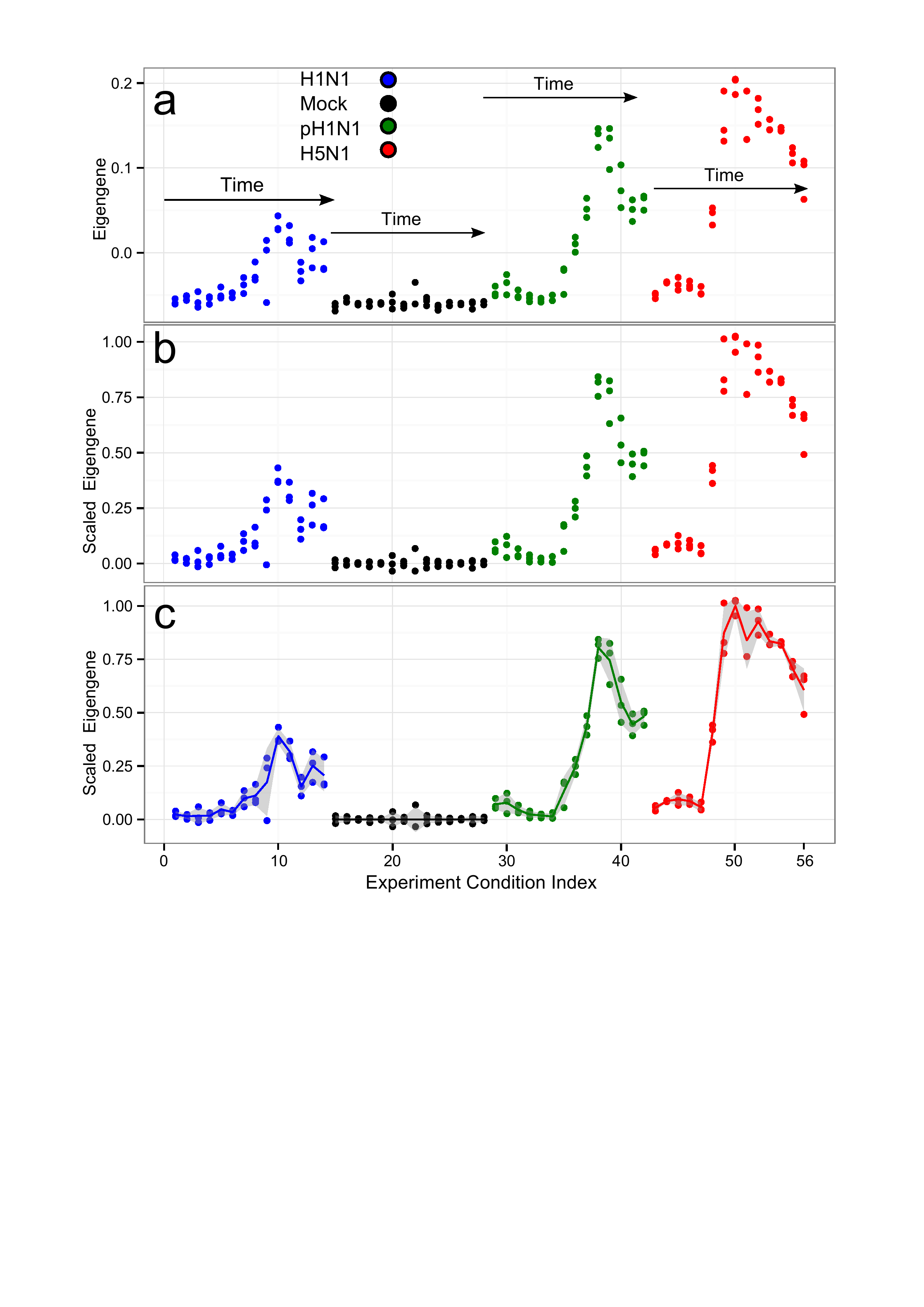

Supplement: S1 Fig — The eigengene is the first principle component (equivalently the first eigenvector) of a matrix of gene expression data. In clustered data in which all genes are highly correlated (as in the case of WGCNA), the eigengene is a scaled approximation of how gene expression changes for all genes in a module (i.e. cluster) across experimental conditions. The eigengene of module N1 is shown in (a) for the 56 experimental conditions considered in this study (4 infection conditions and 14 time points). Each point represents data from a single animal in each experimental condition (3 animals per condition except for H5N1-infected animals at 3 hpi which had 2; total of 167 samples). While the eigengene illustrates the changes in gene expression of module member genes, it is difficult to interpret the changes with respect to the control data. We therefore scaled the eigengene by subtracting from time-matched data the average of the eigengene from mock-infected animals and then dividing by the highest average eigengene for any experimental condition. The scaled difference of the eigengene (SDE) is shown in (b). The SDE now represents the fraction of greatest gene expression (i.e., the fraction of the largest log fold change in gene expression observed between all time-matched, infected-to-control samples). For example, the SDE peaks in experimental condition 50 (H5N1-infected animals at 30 hpi), corresponding to the condition in which the log fold change was greatest for N1 member genes (see Fig 3). The SDE for pH1N1-infected animals at day 3 pi (condition 40) is ~0.5. We would expect the log fold change to be approximately half of that observed in H5N1-infected animals at 30 hpi (see the heatmaps in Fig 3). For completeness, we overlay the mean (lines) and the standard deviation (shaded regions) of the SDE (see panel c) to replicate the results shown in Fig 3. (TIF) [file ppat.1004856.s001.tif]

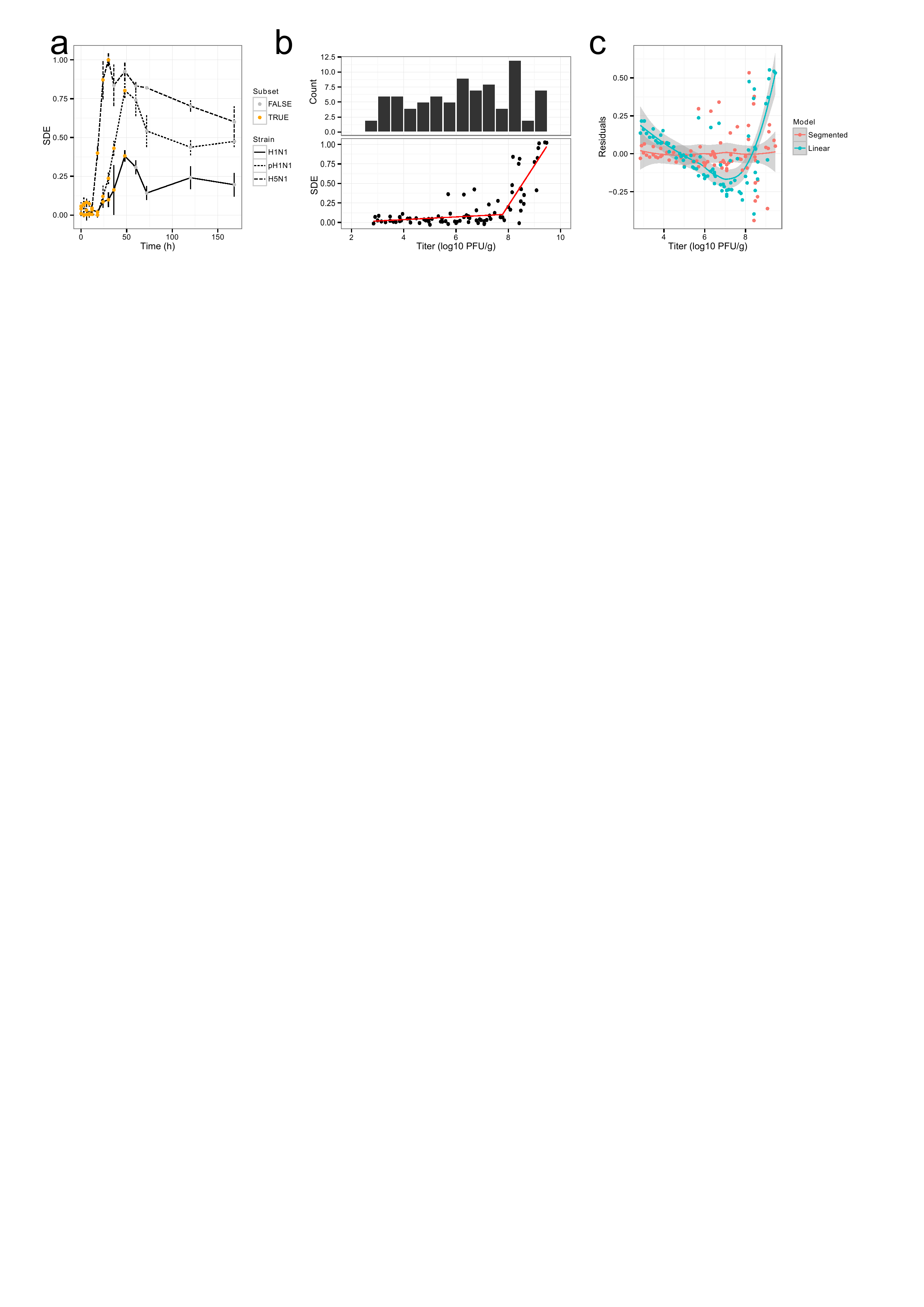

Supplement: S2 Fig — To create the segmented linear model to describe the relationship between the N1 module eigengene and the virus titer data, we selected all titer data that occurred prior to and included the peak of the eigengene and then scaled the data such that the eigengene was bound between [0,1]. The model was then fit to the scaled data. Panel (a) shows the time points selected as training data from each infection data set (indicated by the orange dots); and panel (b) shows the number of data points available for different ranges of the virus titer (top) and the segmented model's fit to the training data (bottom). In panel (c), we used the residuals (i.e., the difference between the predicted values and the actual values) to compare the SLM’s accuracy to that of a simple linear model. The line is the running average and the shaded region is the 95% confidence interval of the mean. The mean of the residuals of the segmented model was always near zero for the full range of virus titers and, thus, is a better fit than the linear model. (TIF) [file ppat.1004856.s002.tif]

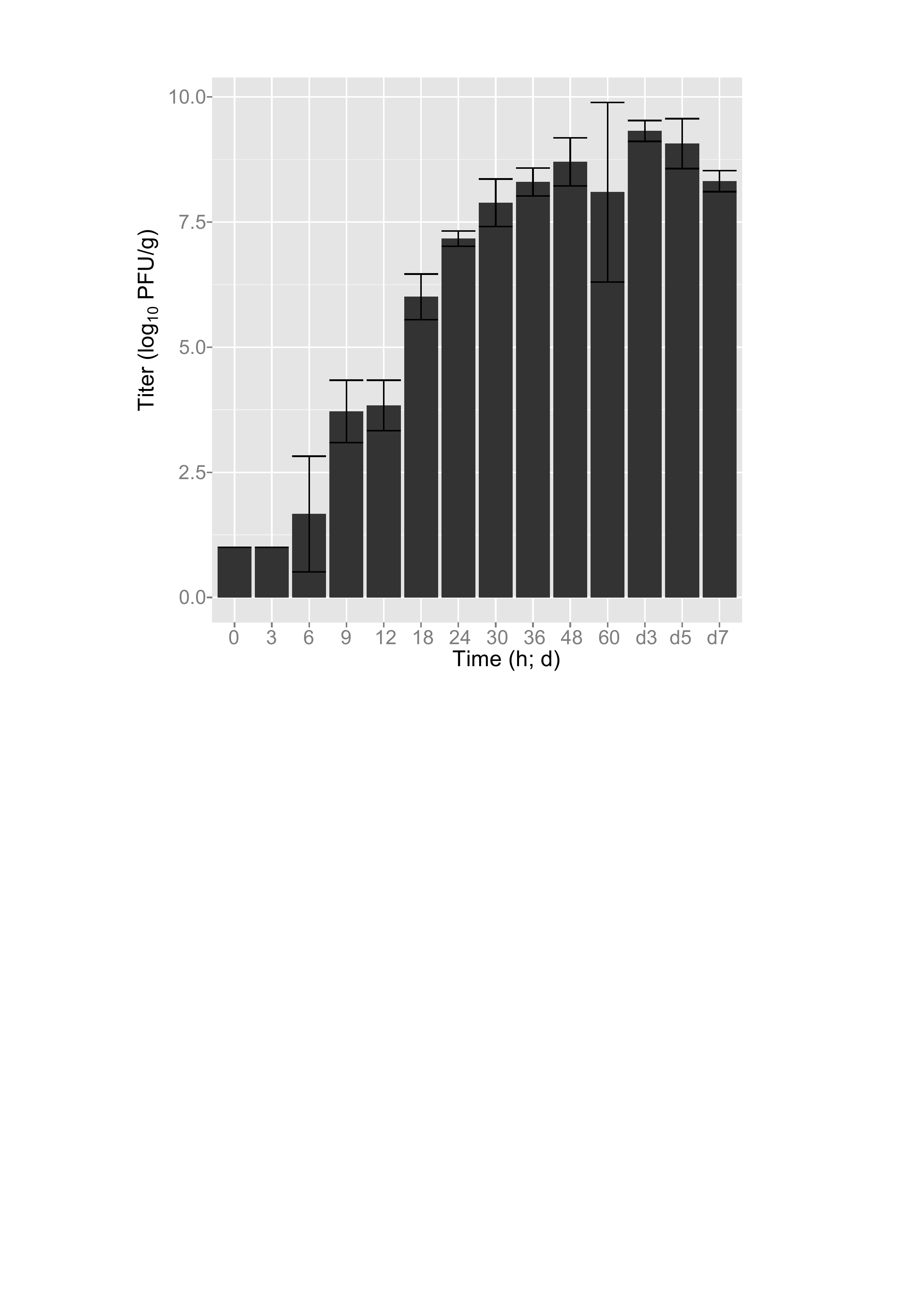

Supplement: S3 Fig — To determine whether the threshold model could predict N1 module gene expression, mice were infected with 103 PFU of the H5N1 virus and lung tissues were harvested for virus titration and gene expression microarray analysis at the same time points used for the original experiment (3 mice per infection group and time point). For each time point, the mean and standard deviation of the virus titer is shown. (TIF) [file ppat.1004856.s003.tif]

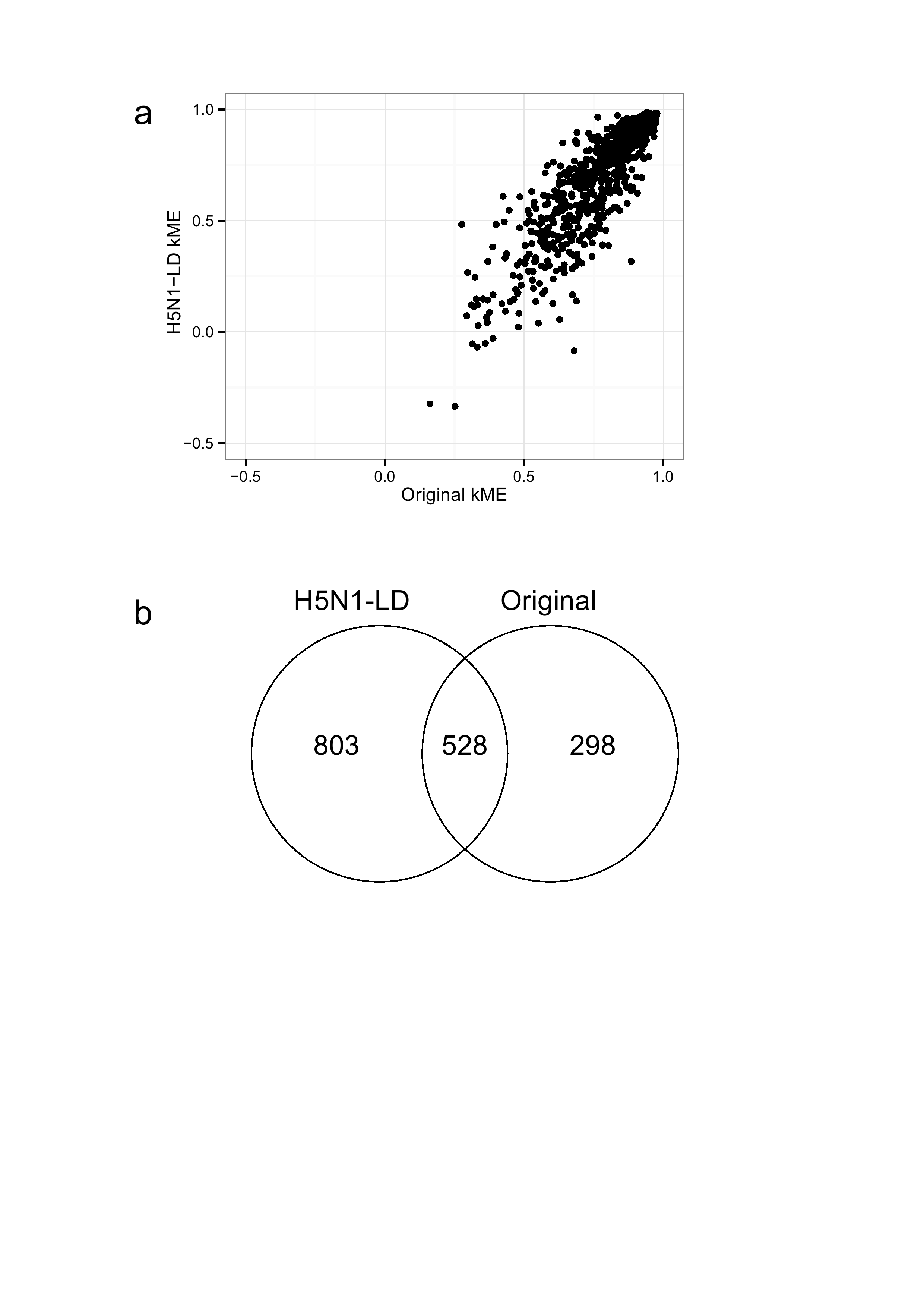

Supplement: S4 Fig — Two procedures were applied to determine whether the N1 genes that we identified as co-expressed in the original clustering analysis were also co-expressed in tissue from mice infected with 103 PFU of H5N1 virus. (a) Compares the correlation between all 1,021 N1 gene transcripts and the eigengene (referred to as the kME) in the original gene expression data (105 PFU infection conditions (referred to as ‘105 PFU’)) and the 103 PFU infection condition (referred to as ‘103 PFU’). More than 90% of the genes exhibited a kME > 0.9. (b) The WGCNA algorithm was repeated with all differentially expressed genes (FDR-adjusted P-value < 0.01 and fold change > 2 for at least 1 time point) from the 103 PFU H5N1 virus infection, and then the Fisher's exact test was used to identify modules enriched for N1 genes. Of the modules in the newly constructed co-expression network, only one module was enriched with the N1 transcripts. Specifically, of the 826 genes originally assigned to N1, 528 (Benjimini-Hochberg-adjusted P-value < 2.2E-16) were differentially expressed and assigned to the same module in the 103 PFU infection condition, as illustrated by the Venn diagram. (TIF) [file ppat.1004856.s004.tif]

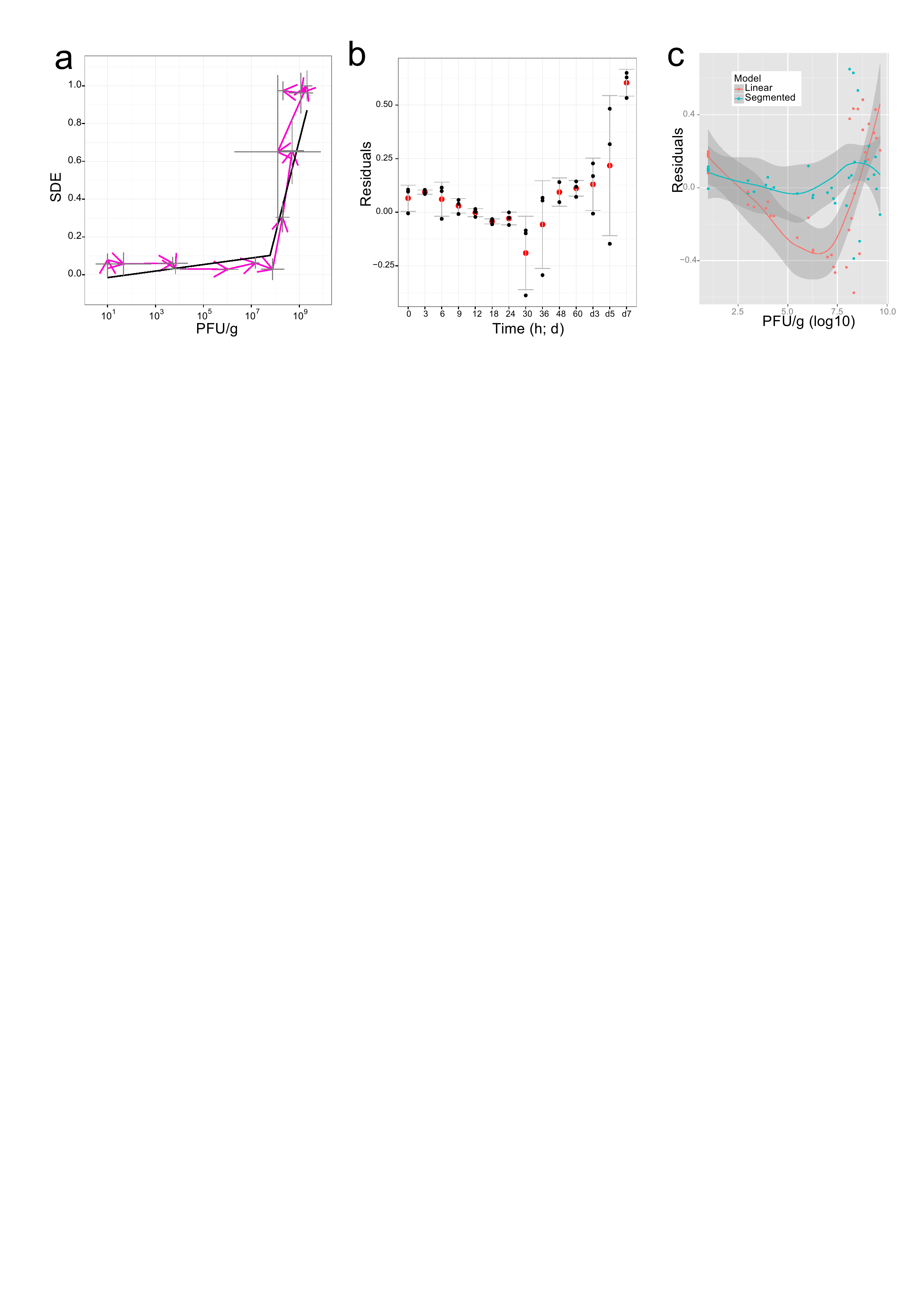

Supplement: S5 Fig — The H5N1-103 PFU eigengene was constructed using the same set of probes assigned to the N1 module from the 105 PFU infection condition and scaled to between [0,1]; and then the segmented linear model trained to the 105 PFU H1N1, pH1N1, and H5N1 data was used to predict H5N1-103 PFU scaled eigengene values. Panel (a) shows a comparison of the predicted (black) and actual (pink arrows depicting the temporal evolution of the gene response) eigengenes, with error bars illustrating the standard deviation of the eigengene and of the log10 of the virus titer. Panel (b) shows how the prediction residuals are distributed over time. For each time point, individual data points (black points) are shown, as well as the average (red points) and standard deviation (gray bars). The greatest deviations occurred at d5 and d7, which was expected as the model was designed only to predict the onset of gene activation and the peak gene expression (peak of the eigengene). On days 5 and 7 post-infection, both the virus titers and gene expression has already peaked and are declining. Panel (c) shows how the prediction residuals are distributed across the spectrum of observed virus titers, as compared to a linear model directly fit to the H5N1-103 PFU N1 eigengene. Individual residuals are indicated by points, and the running average and the 95% confidence intervals are shown by the colored lines and the gray shading, respectively. As for the 105 PFU infection condition, the segmented model performed well across the entire virus titer spectrum, and was significantly better than a linear model fitted directly to the data. (TIF) [file ppat.1004856.s005.tif]

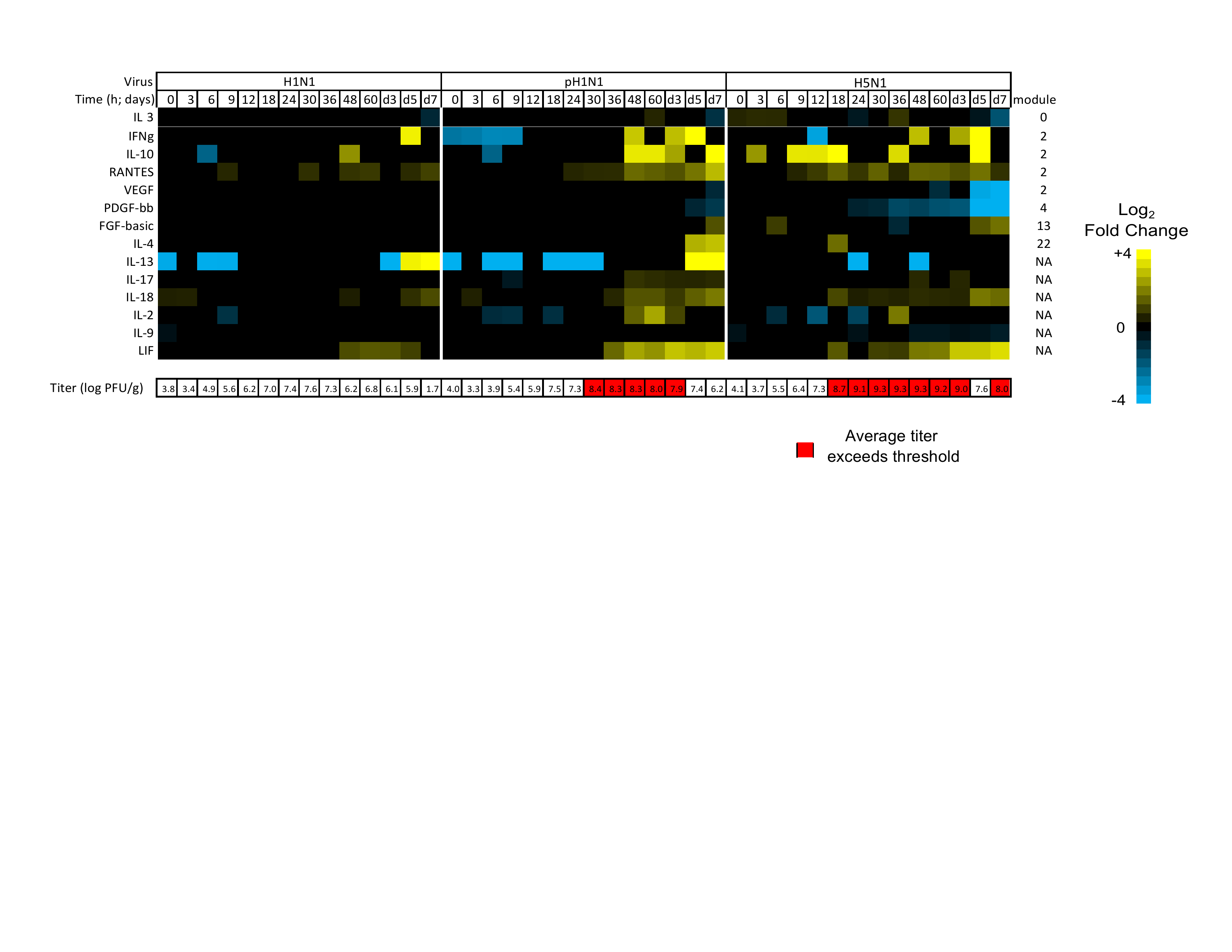

Supplement: S6 Fig — As described in the Fig 5 legend, cytokine concentrations were assayed in the lungs of mice infected with 105 PFU of H1N1, pH1N1, and H5N1 and compared with those in lung tissues from mock-infected animals. This heat map illustrates protein expression values for non-N1-associated cytokines (14 cytokine expression profiles are shown; IL-12(p70)(78) was not detected at any time point and is not included), with a blue-to-yellow scale indicating expression levels (see the key to the right of the panel). The module to which the protein's mRNA transcript was assigned during clustering is shown on the right hand side of the heat map (proteins whose gene transcripts were not DE are labeled ‘NA’), and the average virus titers are shown below the heat map (red indicates that titers exceeded the threshold concentration predicted by the segmented linear model). While IL-18 and leukemia inhibitory factor (LIF) exhibited protein expression patterns consistent with N1 module behavior, the transcripts mapping to these proteins were not differentially expressed and were excluded from the co-expression network construction. (TIF) [file ppat.1004856.s006.tif]

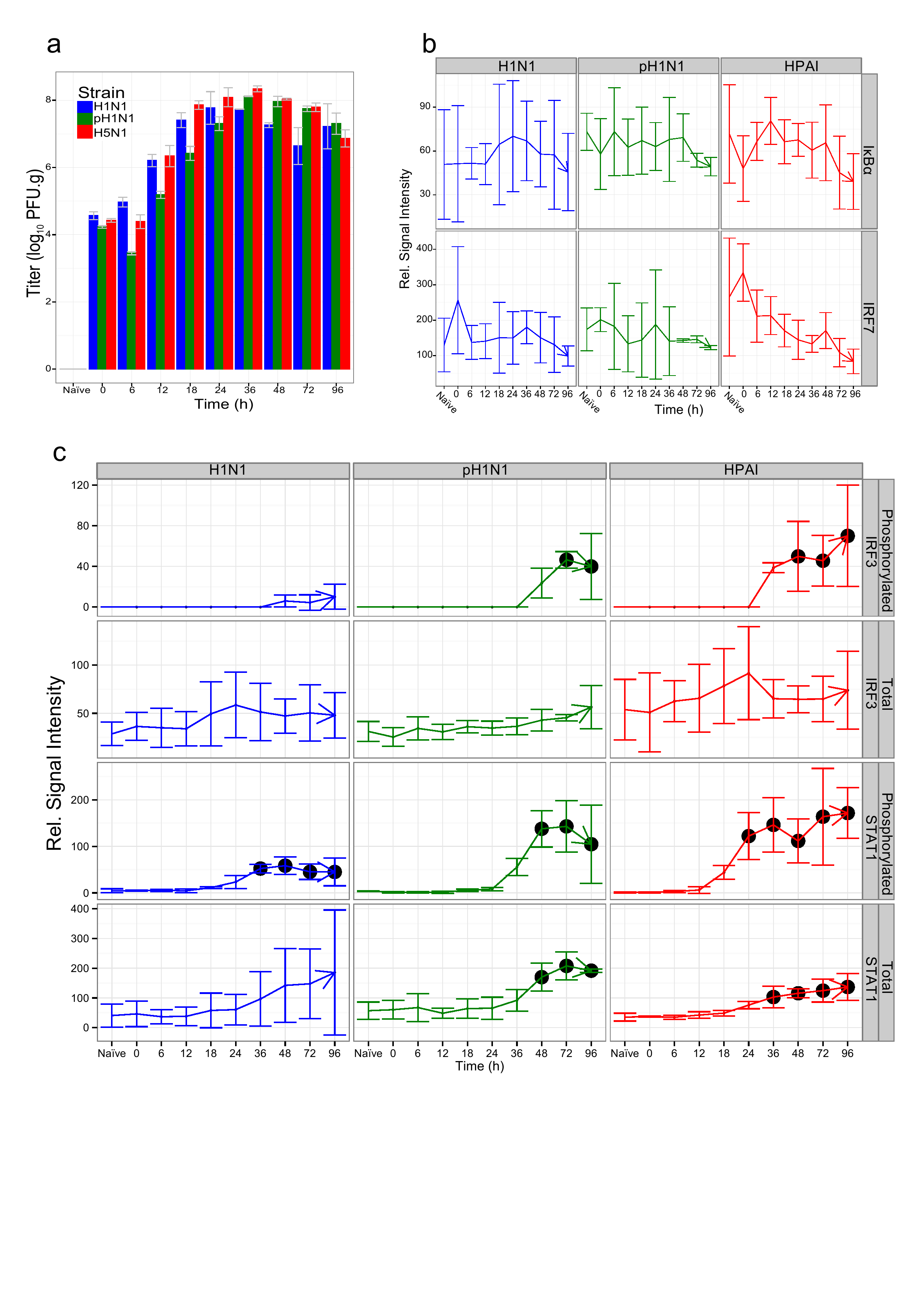

Supplement: S7 Fig — As described in the Fig 5 legend (panel b), an additional set of mice was infected with 105 PFU of H1N1, pH1N1, or H5N1 virus to determine total and phosphorylated levels of transcription factors by means of immunoblotting. Here, virus titers (with standard deviation indicated by gray bars) for each infection condition are shown in panel (a), and immunoblot results are shown in panels (b) and (c). For immunoblot analyses, relative protein concentrations were determined by calculating the ratio of the gray intensity of the measured protein (IκBα, total IRF7, total IRF3 [‘IRF3’], phosphorylated IRF3 [pIRF3’], total STAT1 [‘STAT1’], and phosphorylated STAT1 [‘pSTAT1’]) relative to the gray intensity of actin (i.e., the loading control; referred to as the relative signal intensity, RSI) in each tissue sample. A linear model was used to compare the mean RSI of each protein at each time point to the mean RSI measured in uninfected animals (referred to as ‘naïve’), and a significant difference was defined as having a false discovery rate (FDR) < 0.05. The mean RSI of total IRF3, IRF7, and IκBα did not significantly deviate from the naïve data, but pSTAT1, pIRF3 and total STAT1 significantly differed from the naïve data at several time points (significant distinct time points are indicated by large, black dots). (TIF) [file ppat.1004856.s007.tif]

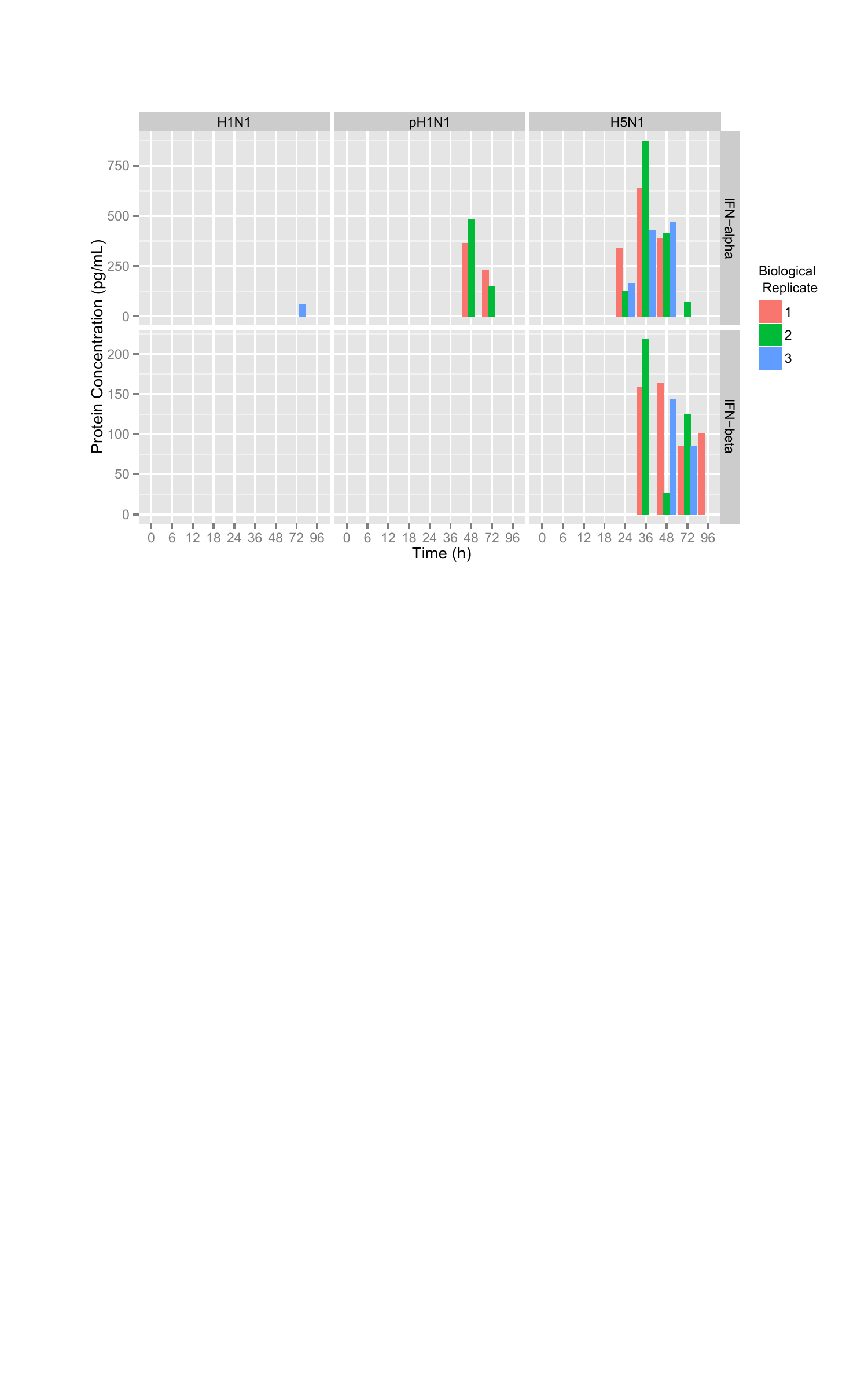

Supplement: S8 Fig — As described in the Fig 5 legend (panel b), mice were infected with 105 PFU of H1N1, pH1N1, or H5N1 virus to determine IFN protein concentrations. The protein concentration of IFN-α/β proteins for each replicate at each time point is shown. (TIF) [file ppat.1004856.s008.tif]

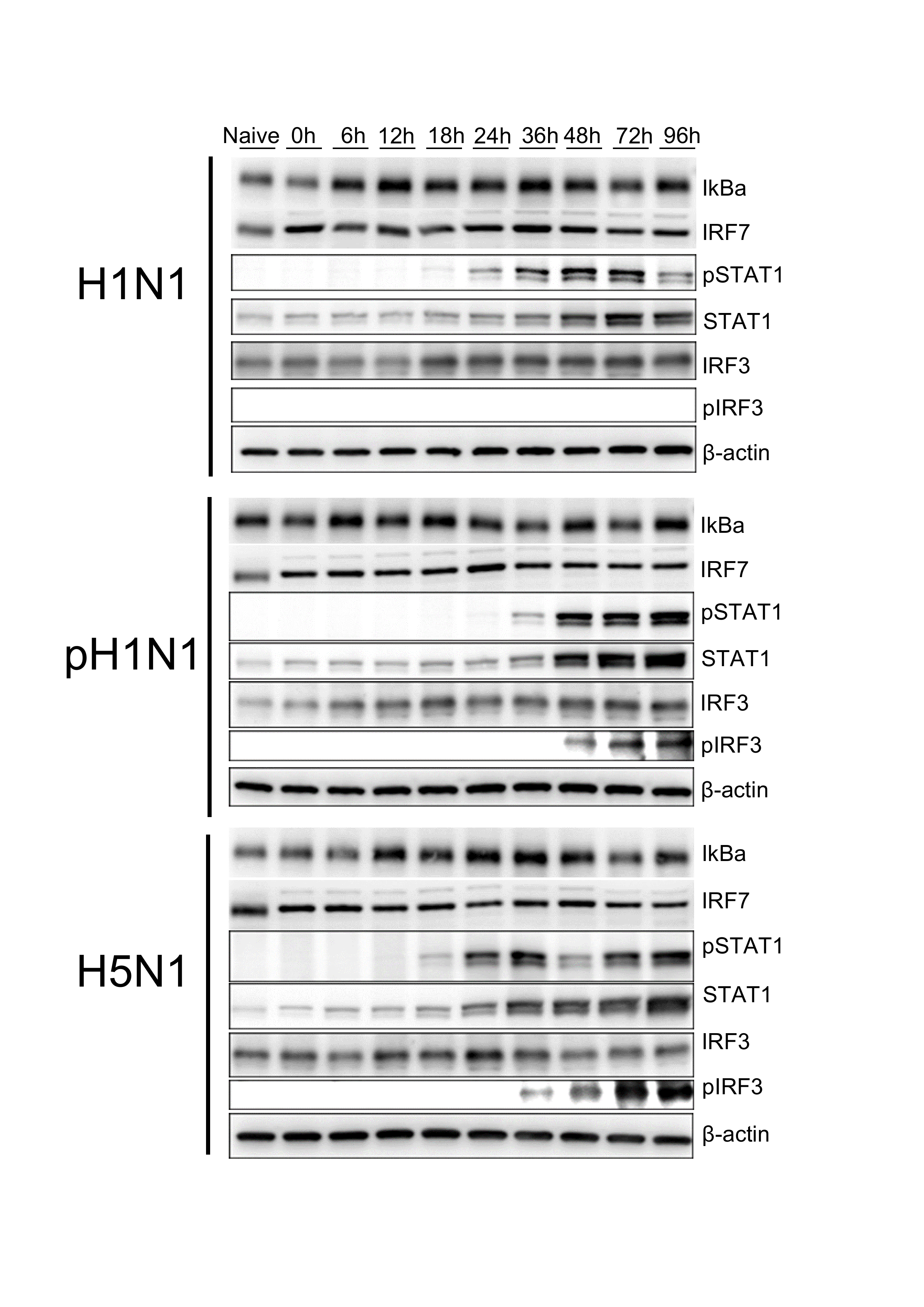

Supplement: S9 Fig — As described in the Fig 5 legend (panel b), mice were infected with 105 PFU of H1N1, pH1N1, or H5N1 virus. Representative images of Western blots of lung homogenates from 3 mice per infection per time point are shown. (TIF) [file ppat.1004856.s009.tif]

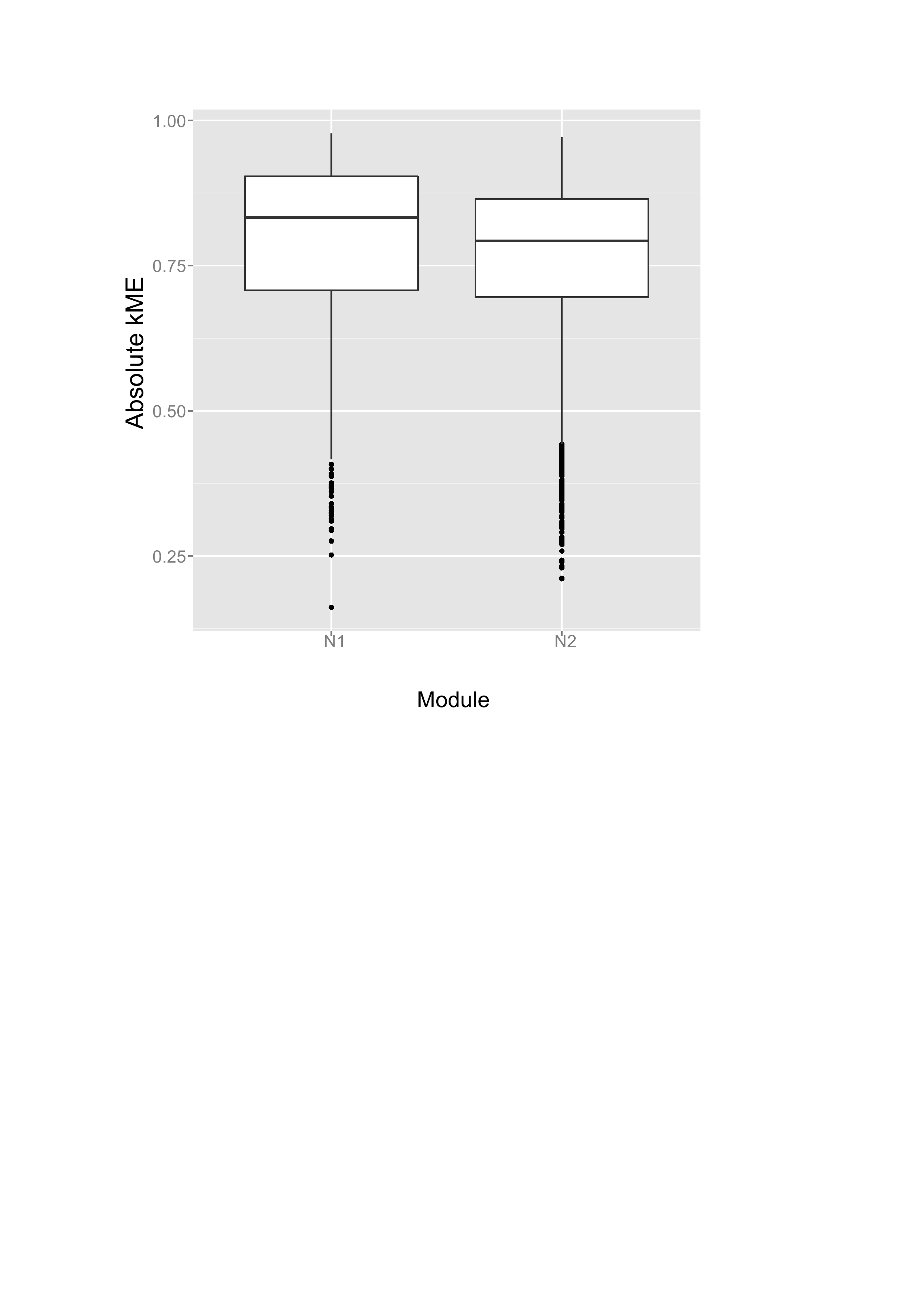

Supplement: S10 Fig — The intramodular correlation (kME or correlation between each transcript and the eigengene) can be used to assess clustering quality. We show boxplots to illustrate the distribution of the kMEs for all transcripts belonging to the N1 and N2 modules. (TIF) [file ppat.1004856.s010.tif]

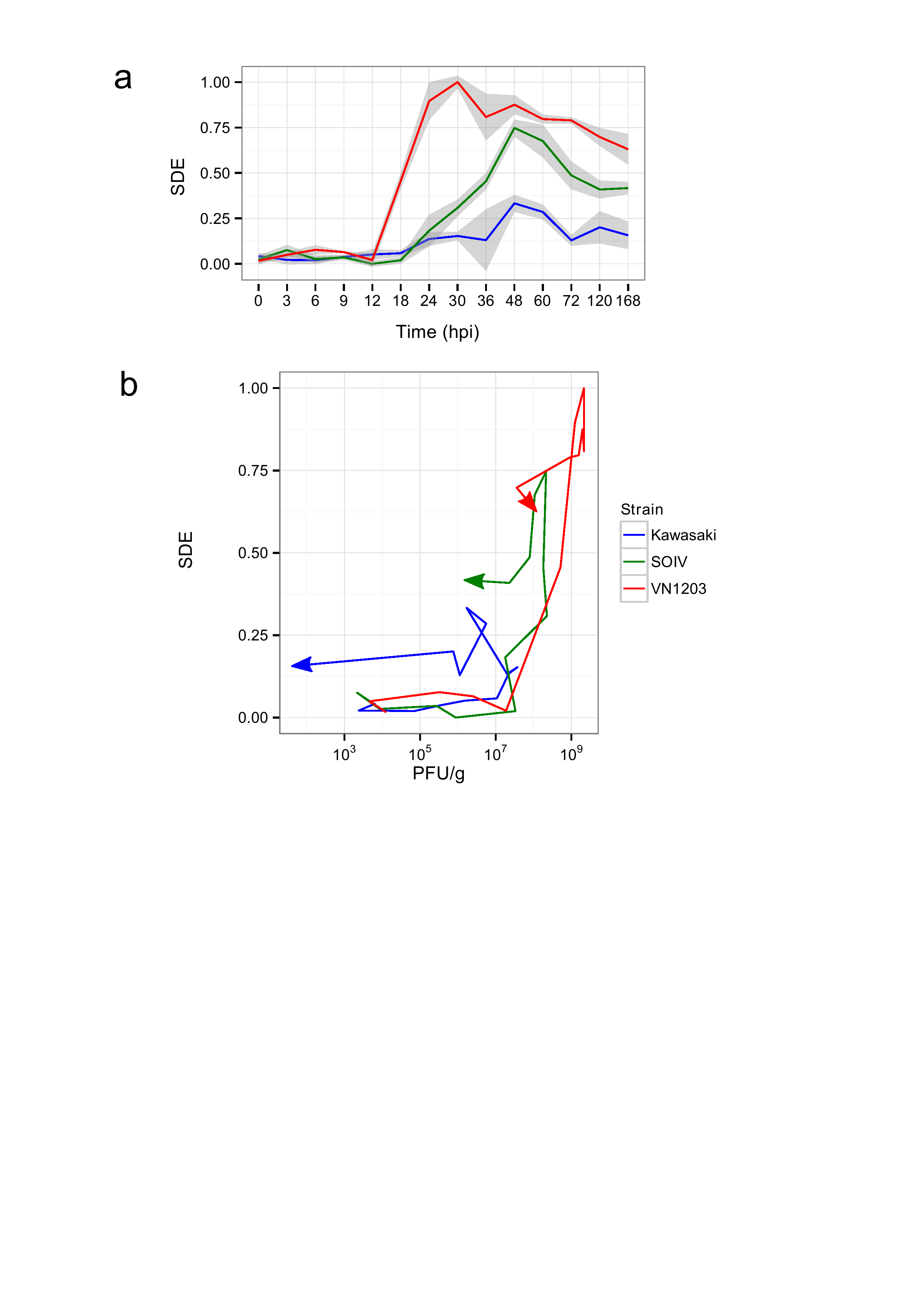

Supplement: S11 Fig — The N1 module presented in this work was identified by developing an unsigned co-expression network and then focusing on the kME- and kME+. Therefore, the N1 kME+ set of genes should also cluster when developing a signed co-expression network. We confirmed this by repeating the WGCNA procedure for signed network (power = 14). Of the N1 kME+ transcripts, 93.2% were assigned to the same cluster in the signed co-expression network (the new module is referred to as N1signed). Furthermore, 90.9% if the N1signed transcripts were N1 kME+ transcripts. We then confirmed that the same eigengene dynamics were observed. The scaled difference of the eigengene (SDE) of the N1signed module is shown versus time (a) and versus virus titers (b). (TIF) [file ppat.1004856.s011.tif]
